# The multilevel organismal diversity approach deciphers difficult to distinguish nudibranch species complex

**DOI:** 10.1038/s41598-021-94863-5

**Published:** 2021-09-15

**Authors:** Tatiana A. Korshunova, Floor M. F. Driessen, Bernard E. Picton, Alexander V. Martynov

**Affiliations:** 1 Koltzov Institute of Developmental Biology RAS, 26 Vavilova Str., 119334 Moscow, Russia; 2 Bureau Waardenburg BV, Aquatic Ecology, Varkensmarkt 9, 4101 CK Culemborg, The Netherlands; 3 Royal Netherlands Institute for Sea Research (NIOZ), PO Box 59, 1790 AB Den Burg, The Netherlands; 4 National Museums Northern Ireland, Holywood, Northern Ireland BT18 0EU UK; 5 Queen’s University, Belfast, Northern Ireland UK; 6 Zoological Museum, Moscow State University, Bolshaya Nikitskaya Str. 6, 125009 Moscow, Russia

**Keywords:** Evolution, Zoology

## Abstract

Species identification is a key procedure for broad-scoped ecological, phylogeographic and evolutionary studies. However, to perform a taxonomic study in the molecular era is a complicated task that has many pitfalls. In the present study we use particular examples of common but difficult to distinguish European species within the genus of *Polycera* (Nudibranchia, Mollusca) to discuss the general issues of the “cryptic species” problem that has broad biological and interdisciplinary importance and can significantly impede ecological, evolutionary, and other biodiversity-related research. The largest dataset of molecular and morphological information for European nudibranchs ever applied encompasses a wide geographical area and shapes a robust framework in this study. Four species are recognized in the species complex, including a new one. It is shown that a lack of appropriate taxonomic analysis led recently to considerable errors in species identity assessment of this complex. Chromatic polymorphism for each species is mapped in a periodic-like framework and combined with statistical analysis of the diagnostic features that considerably facilitates identification of particular species in the complex for biologists and practitioners. The present study evidently shows that “cryptic” and “non-cryptic” components are present within the same species. Therefore, this species complex is well suited for the exploring and testing of general biological problems. One of the main conclusions of this study is that division of biological diversity into “cryptic” and “non-cryptic” components is counterproductive. We propose that the central biological phenomenon of a species can instead be universally designated as *multilevel organismal diversity* thereby provide a practical set of methods for its investigation.

## Introduction

The species concept is a central biological problem^1–4^. Polymorphism became a key notion for a species concept in the mid-twentieth century^5,6^. The problem of a hidden species (when among polymorphic lineages several difficult-to-distinguish but separate species do exist) had already emerged in the pre-molecular phylogenetic era^7^. When application of molecular data became a routine, any potentially hidden lineages were labelled as “cryptic species”^8^. Recently several studies independently showed that this counterproductively disrupts organismal diversity into “cryptic” and “non-cryptic” species^9–12^.

Particularly, to attempt to make a distinction between ‘non-cryptic species’ and ‘cryptic/sibling’ species we need a number of intermediate terms, which could grow indefinitely^12^. However, despite that, there are continuous recent attempts to putatively distinguish the terms “cryptic” and “non-cryptic” species, commonly with the presentation of some morphological distinguishing characters that, by definition, mean that the newly separated species cannot be considered as “cryptic”^13–15^. Hidden diversity therefore is an important problem, especially in the context of the polymorphism^16,17^, which can appear as a parallel-like pattern of characters in two or more closely related species^18,19^. Because within already fine-scale delineated species, it is still possible to uncover more diversity^12^, the commonest European nudibranchs *Polycera* are investigated in this study in order to further test the reliability of the currently widely discussed “cryptic species” concept*.* It was specially highlighted in a recent study that the “cryptic species problem” represents a significant importance for the most general species problem^20^ and therefore needs a further detailed exploration.

The species of the genus *Polycera* are also important for broad-scoped evolutionary and ecological studies, in environmental monitoring, conservation, and are well known to the researchers from various fields and practitioners^21–25^. Particularly, *Polycera* species became the focus of a study with broad interdisciplinary importance of evolution of the warning colouration^26^. Furthermore, a *Polycera* species already became an indicator of global warming since it is among few species of European nudibranch which was recently found in the Arctic Barents Sea, but importantly, it was never indicated in historical records for this area^27^. Climate changes are among key modern challenges for protection of the world biodiversity^28^. Therefore this species complex is very well suited for the broad-scaled exploring and testing of many biological problems, including the key cryptic species problem because on one hand this is a very common marine organism which inhabits the whole European region, but on the other hand the species within this complex exhibit substantial polymorphism, which is crucial for study of the general species problem.

In the present study the largest molecular dataset ever available for European nudibranchs at species level is used (in total ca. 200 specimens). The present dataset encompasses a broad area geographically from the subarctic regions of Northern Europe to the Mediterranean Sea in the south and includes data from several European countries and is comprehensive in coverage of morphological, molecular and geographic parameters. It allowed us to exhaustively investigate potential “hidden lineages” within a given species complex and align it with the fine-scale morphological characters. This study also considerably illuminates the problem where an immense external polymorphism is nevertheless restrained by the presence of internal well-diagnosable characters^29^. Ideally, however, a species can be distinguished and diagnosed using external features. This is especially important for quick identification in the field during ecological monitoring. The amount of molecular data presented here further allows mapping of the big range of the polychromatic diversity into several periodically arranged chromatic variants. Afterwards, using statistical methods, the range of variability of the key diagnostic characters are checked inside of these chromatic variants. Thus, a broad integration of molecular and morphological data as well as phylogenetic, phylogeographical and ecological patterns, and statistical analysis will be used in the present study. Application of this approach enables the presentation of diagnosable taxonomic units (important for broad-scoped ecological, phylogeographic and evolutionary studies) in a more efficient way than it is employed currently, and therefore shows that the used distinction between “cryptic” and “non-cryptic” diversity is an artificial one.

## Materials and methods

### Sampling

Material for this study was obtained by scuba diving at widely separated locations in Europe: Denmark, Germany, Ireland, the Netherlands, Norway, Spain, Sweden, Portugal, and the United Kingdom. Specimens were photographed underwater and measured. Magnesium sulphate (7%) has a relaxing effect on muscles, therefore each specimen was immediately stored in a dilution with seawater (1:1) at 4 °C overnight, before preserving in 96% ethanol. The specimens were deposited in the Zoological Museum of Lomonosov Moscow State University (ZMMU), National Museums Northern Ireland and in Bureau Waardenburg BV, Aquatic ecology, the Netherlands (FD).

### Nomenclatural acts

The electronic version of this article in Portable Document Format (PDF) will represent a published work according to the International Commission on Zoological Nomenclature (ICZN), and hence the new name contained in the electronic version is effectively published under that Code from the electronic edition alone. This published work and the nomenclatural acts it contains have been registered in ZooBank, the online registration system for the ICZN. The ZooBank LSIDs (Life Science Identifiers) can be resolved and the associated information viewed through any standard web browser by appending the LSID to the prefix http://zoobank.org/. The LSID for this publication is: [urn:lsid:zoobank.org:pub:15279E86-D7E2-4765-BF22-C0EB01E48E3D]. The online version of this work is archived and available from the following digital repositories: PubMed Central and CLOCKSS.

### Morphological analysis

The external and internal morphology was studied using a stereomicroscope and digital cameras (Nikon D-810, Nikon D-7000, Nikon D-600 and Nikon D-80). The buccal masses were extracted and processed in 10% sodium hypochlorite solution to extract the radula and the jaws. Reproductive systems were examined using the stereomicroscope. The jaws were analyzed under a stereomicroscope and then photographed. The radulae and jaws were rinsed in water and 70% ethanol, then dried, mounted on stubs using carbon tape, coated with gold and palladium and finally examined using scanning electron microscopes (CamScan Series II and JSM 6380). The images were captured using a maximum quality mode (4) in CamScan II and a 80-s capturing mode in JSM 6380.

### Statistical analysis

External morphology features (body length; number of frontal veil appendages, rhinophoral lamellae, and gills) were evaluated statistically using nonparametric Mann–Whitney rank sum test.

### Molecular analysis

A total of 102 *Polycera* specimens of various colour patterns and one *Palio dubia* were successfully sequenced in the Netherlands and Russia for the mitochondrial cytochrome c oxidase subunit I *(COI) gene,* and the ribosomal *16S RNA gene* (see Supplementary information S1 for DNA extraction procedure, and PCR amplification options)*.* All new sequences were deposited in GenBank (Supplementary information, Table S1, highlighted in bold). Original data and publicly available sequences were aligned with the MAFFT algorithm^30^. Separate analyses were conducted for COI (658 bp), 16S (463 bp), and concatenated data (1121 bp). Evolutionary models for each data set were selected using MrModelTest 2.3^31^. The GTR + I + G model was chosen. Two different phylogenetic methods, Bayesian inference (BI) and Maximum Likelihood (ML), were used to infer evolutionary relationships. Bayesian estimation of posterior probability was performed in MrBayes 3.2^32^. Four Markov chains were sampled at intervals of 500 generations. Analysis was started with random starting trees and 5 × 10^6^ generations. ML analysis was performed using RAxML 7.2.8^33^ with 1000 bootstrap replicates. Final phylogenetic tree images were rendered in FigTree 1.4.2. To evaluate the genetic distribution of the different haplotypes, a haplotype network for the COI molecular data was reconstructed using Population Analysis with Reticulate Trees (PopART, http://popart.otago.ac.nz) with the TCS-network method. The program Mega7^34^ was used to calculate uncorrected p-distances between all sequences. Additionally, Automatic Barcode Gap Discovery (ABGD)^35^ was used to group the sequence data into operational taxonomic units. The alignment from the COI marker for all *Polycera* specimens under consideration was submitted and processed in ABGD using the Jukes–Cantor (JC69) and Kimura (K80) models with default settings.

### Building the periodic-like rows of chromatic variants

**Figure 1:**
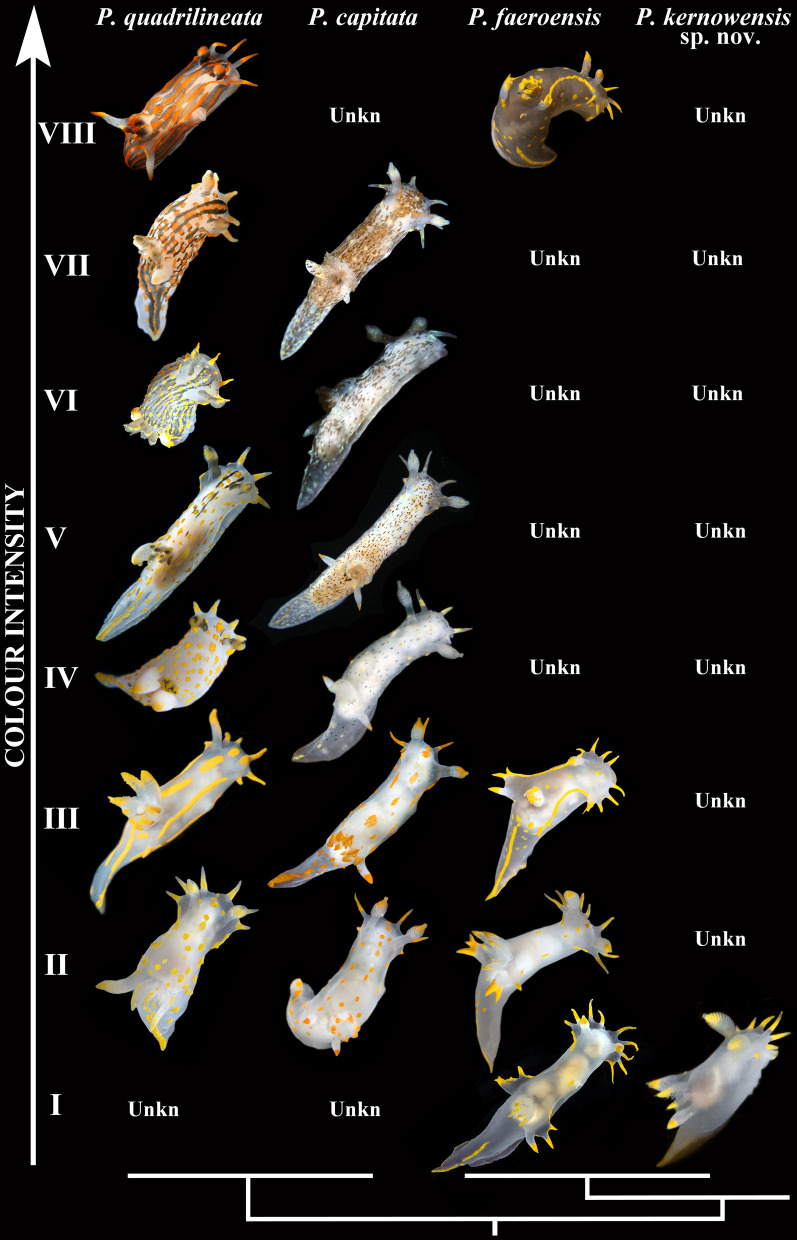
Periodic-like presentation of chromatic variation patterns among species within European *Polycera*, represented as vertical rows. Eight main periods (horizontal rows, roman numerals) are presented whereas spotless body/colourless forms are at the bottom and forms with a maximal number of spots/coloured are at the top. Non-observed forms for each particular species are indicated as “unkn” = “unknown”.

Similar chromatic variants within each potential species were aligned using calibration by the degree of light to dark surface pigmentation and transparency of body tissue, including presence of black stripes and brownish/dark pigmentation on the dorsal and lateral sides of the body. These similar forms establish horizontal rows of similar looking specimens within the species (Fig. 1). In total, eight chromatic variants (horizontal rows I–VIII) are recognized: I—body semi-transparent white, orange–yellow spots or brownish/dark colouration completely absent on the dorsal and lateral sides (orange–yellow colouration restricted to the frontal veil appendages, tips of rhinophores, gills, and postbranchial lobes); II—body semi-transparent white, few or indistinct orange–yellow spots present on the dorsal and lateral sides; III—orange–yellow (sometimes with reddish hue) spots are distinct and tend to form lines; IV—in addition to orange–yellow spots blackish or brownish spots or weak lines appeared (in case of lines, mostly in the anterior part of the body); V—in addition to orange–yellow spots, blackish stripes appear (but do not form continuous lines throughout dorsal side), blackish/brownish spots become evident and closer each other; VI—blackish stripes become evident and form continuous lines throughout dorsal side, blackish/brownish spots begin to blend together and form faint stripe-like pattern, orange–yellow spots evident in striped morphs, and less evident or almost absent in spotted morphs; VII—blackish stripes or a brownish (with a greenish hue) colouration pattern become dominant, orange–yellow spots are distinct in striped morphs, and less distinct or almost absent in spotted morphs. VIII—the blackish stripes pattern remains evident or merged, whereas orange–yellow spots (often with more intense reddish colouration) are merged into distinct lines. The proposed scheme of the chromatic variants has a biological basis since it was shown for *P. quadrilineata* s.l. that during the earlier post larval ontogenetic development orange and blackish colouration is weak or almost absent, and distinct spots, lines and stripes appear only towards later ontogenetic stages^36^ and this coincides well with general patterns of the ontogeny among dorid nudibranchs, when colourless or white forms appear first, and intense colouration is added later.

## Results

### Morphological and statistical analyses

**Figure 2:**
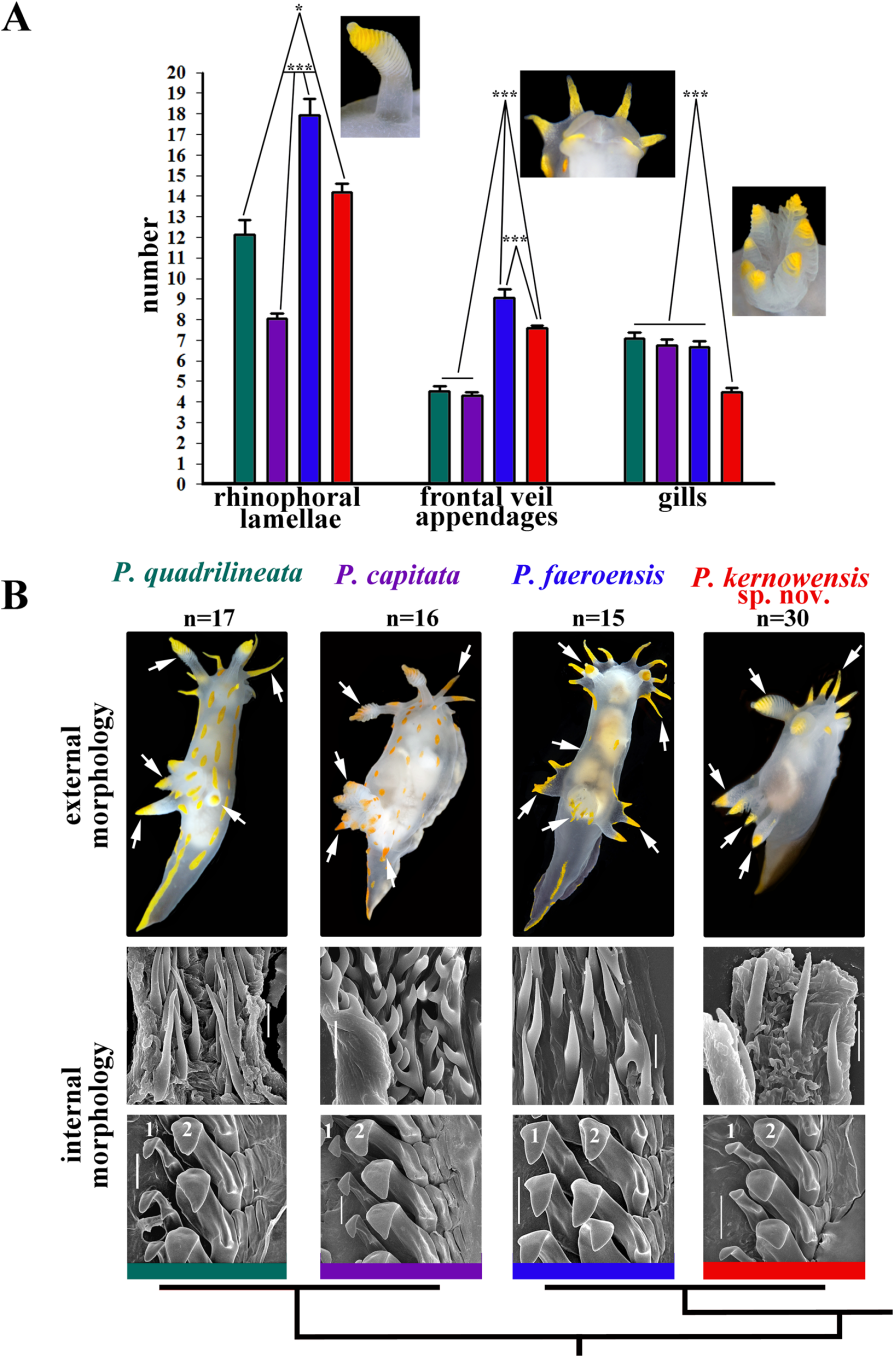
Statistical analysis (**A**) of the external diagnostic characters (indicated by arrows) among white and orange colour variants of detected species within European *Polycera* (mean ± the standard error of the mean), and presentation of the internal diagnostic features (**B**)*.* Scale bars: all radulae – 100 μm, all spines − 10 μm.

The scheme of description of the chromatic polymorphism among the monophyletic group of closely related species of *Polycera* which inhabit European waters is consistently applied to each of the four potential species in this complex (Fig. 1, vertical columns). Among specimens whose external and internal morphology correspond to the descriptions of *Polycera quadrilineata* and *P. capitata*, specimens with the colour patterns which correspond to the rows II–VIII were detected (Fig. 1, horizontal rows show eight different color patterns). In *P. faeroensis* specimens with colour patterns for the rows I–III and VIII were found, whereas for *P. kernowensis* sp. nov. (holotype ZMMU Op-755, ZooBank registration: urn:lsid:-zoobank.org:act: 5C821EFD-FB12-49D5-A9C3-026A325F6D21) specimens with colour patterns corresponding only to row I were found. Afterwards, an additional morphological study in order to detect morphological differences between similar specimens of I–III rows (semitransparent white body and absence/presence yellow-orange spots/lines, that was observed in 42% among *P. quadrilineata* specimens, used for statistical analysis, 47% *P. capitata*, 88% *P. faeroensis*, and 100% of *P. kernowensis* sp. nov.) (Fig. 1). In the present study it is shown that these similar white-and-yellow specimens of all four species reveal significant statistical differences in number of rhinophoral lamellae (p = 0.023 between *P. quadrilineata* and *P. kernowensis* sp. nov.; p < 0.001 between all other species). For the number of frontal veil appendages there were no significant statistical differences between *P. quadrilineata* and *P. capitata.* However, such differences were revealed between *P. faeroensis* and *P. kernowensis* sp. nov. (p < 0.001); *P. faeroensis* compared with *P. quadrilineata* or *P. capitata* (p < 0.001); *P. kernowensis* sp. nov. compared with *P. quadrilineata* or *P. capitata* (p < 0.001). There were no significant statistical differences in number of gills for *P. quadrilineata*, *P. capitata,* and *P. faeroensis*. But *P. kernowensis* sp. nov. shows statistically significant fewer gills compared with *P. quadrilineata*, *P. capitata,* and *P. faeroensis* (p < 0.001) (Fig. 2A, Table 1, Table S2). It is unmistakably visible that *P. faeroensis* commonly possesses more than one pair of postbranchial lobes (Fig. 2B). Significant statistical differences in external morphology were revealed for specimens of all colour patterns (Supplementary information, Fig. S1). Mean *P. faeroensis* body length is statistically significantly bigger than mean body length of *P. quadrilineata*, *P. capitata,* and *P. kernowensis* sp. nov. Mean *P. capitata* and *P. kernowensis* sp. nov. body length is statistically significantly smaller than mean body length of *P. quadrilineata* and *P. faeroensis* (p < 0.001, Fig. S1, Table S2)*.* In addition, differences in internal morphology were revealed (Fig. 2B). *P. capitata* have clear differences from the other species in shape of the copulative spines. *P. faeroensis* have clear differences in shape and size of the lateral teeth of radula.

**Table 1:** Verified and updated comparison of key diagnostic characters of the European *Polycera* species complex based on the *multilevel organismal diversity* approach.

	*P. quadrilineata*	*P. capitata*	*P. faeroensis*	*P*. *kernowensis* sp. nov
Length (mm)	Max. 45	Max. 16	Max. 45	Max. 20
Reported chromatic variants	II–VIII	II–VII	I–III, VIII	I
Chromatic differences	Few or numerous blackish stripes in chromatic variants IV–VII	Few or numerous blackish or brownish (with greenish hue) spots present in chromatic variants IV–VII	Chromatic variants IV–VII not known	Chromatic variants II–VIII had not known
Number of frontal veil appendages	Mean 4–5, max. 7	Mean 4–5, max. 6	Mean 8–9, max. 14	Mean 7–8, max. 9
Number of lamellae of rhinophores	Mean 11–12, max. 17	Mean 8, max. 11	Mean 18–19, max. 25	Mean 14–15, max. 22
Number of gills	Mean 6–7, max. 11	Mean 6–7, max. 9	Mean 6–7, max. 11 gills	Mean 4–5, max. 7 gills
Postbranchial lobes	Simple (rarely bifurcated), one pair	Simple, one pair	Compound (2–5 pairs plus smaller tubercles; if simple-looking lobes, several smaller tubercles present in addition)	Simple, one pair
Jaws	With moderately narrow shoulders and strong, distinct wing-like expansions	With moderately narrow shoulders and distinct wing-like expansions, delicate	With broad, rounded shoulders, without distinct wing-like expansions	With moderately narrow shoulders, and distinct wing-like expansions
Reported radula formula	9–20× 0–5.1.1.0.1.1.0–5	9–10× 0–5.1.1.0.1.1.0–5(6?)	11–16× 0–3.1.1.0.1.1.0–3	7–11× 0–4.1.1.0.1.1.0–4
First and second inner lateral teeth	First teeth differ in shape from second one, usually larger	First teeth differ in shape from second one, usually larger	First teeth similar in shape and size to second one	First teeth differ in shape from second one, usually larger
Outer lateral teeth number	Up to 5 (5 is more common)	Up to 5(6?) (4 is more common)	Up to 3	Up to 4
Middle cusp of first lateral teeth	Distinct in majority of radular teeth	Distinct in majority of radular teeth	Not evident	Evident in the posterior rows of radula
Ampulla	Relatively large, conspicuously bent in middle part	Relatively small, somewhat enlarged in the proximal part	Relatively large, conspicuously bent in middle part	Relatively small, not evidently bent in middle part
Bursa copulatrix	Large, elongate	Medium-sized, widened, oval	Very large, elongate	Large, elongate
Vas deferens	Relatively long, distinctly widened distally	Relatively long, not widened distally	Very long, not widened distally	Relatively long, not widened distally
Copulative spines	Needle-shaped, straight or winding or shorter cones	Short hooks or short to elongate cones	Long, straight or slightly bent spines or shorter more elongate cones with a peculiar base with a hole	Elongate, somewhat hooked cones

The characters are given for adults or subadults individuals.

### Molecular phylogenetic analysis

**Figure 3:**
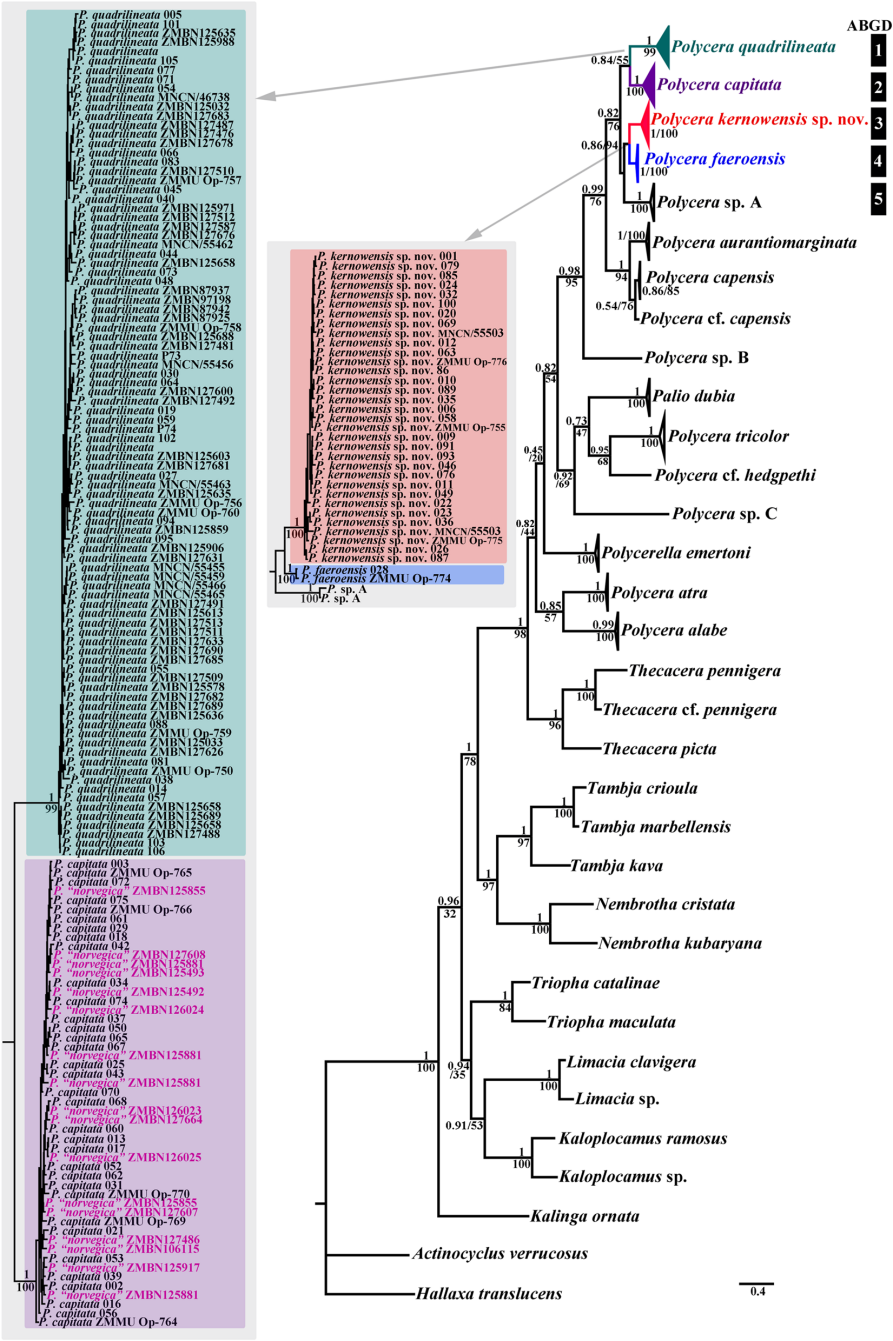
Phylogenetic relationships of *Polycera* specimen based on COI + 16S concatenated dataset inferred with Bayesian (BI) inference. Numbers above branches represent the posterior probabilities from BI. Numbers below branches indicate the bootstrap values for Maximum Likelihood (ML).

**Figure 4:**
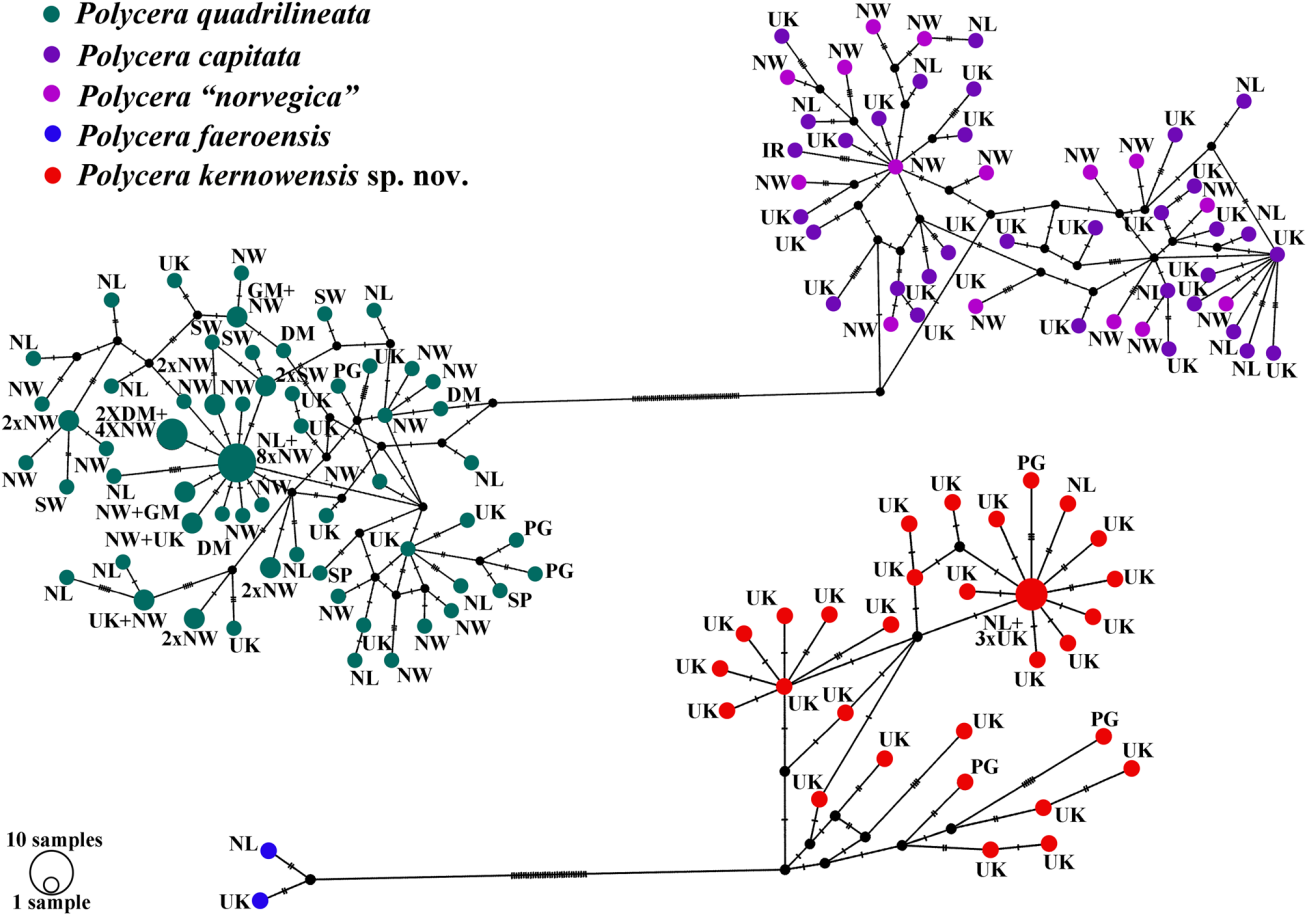
The haplotype network for *Polycera* specimens based on cytochrome c oxidase subunit I (COI) molecular data (642 bp). Phylogeographic data are represented within a broad geographic framework. Each circle represents one haplotype, the circle area indicates the occurrence rate. Each cluster (species) is coloured individually (*P. quadrilineata* in green, *P. capitata* in purple, “*P. norvegica*” in pink, *P. faeroensis* in blue and *P. kernowensis* sp. nov. in red.). *DM* Denmark, *GM* Germany, *IR* Ireland, *NL* Netherlands, *NW* Norway, *PG* Portugal, *SP* Spain, *SW* Sweden, *UK* United Kingdom.

Phylogenetic analysis was performed using 197 specimens of *Polycera*, including data for 178 *P. quadrilineata* species complex (102 of which were preliminarily divided into four groups using the methods described above and data for 76 downloaded from GenBank), and 24 outgroup specimens. The dataset consisted of 356 nucleotide sequences including COI and 16S genes. Bayesian Inference (BI) and Maximum Likelihood (ML) analyses based on the combined dataset for the COI, and 16S genes yielded similar results (Fig. 3). To define species, we use a set of methods^12,37^ including phylogenetic tree topologies, ABGD analysis, pairwise distances and the haplotype network containing phylogeographic data rendered using PopART (Fig. 4, Table 2). The results of this study confidently confirmed the presence of four species among *P. quadrilineata* similar specimens that coincided with species detected before the molecular study: *P. quadrilineata, P. capitata* (that do not show any differences from the recently described *P. norvegica*), *P. faeroensis*, and *P. kernowensis* sp. nov. A clade containing *P. quadrilineata* (n = 92, PP = 1, BS = 99) has the closest position to the clades containing *P. capitata* (n = 34) combined with former *P*. *norvegica* (n = 17, PP = 1, BS = 100)*, P. faeroensis* (n = 2, PP = 1, BS = 100), *P. kernowensis* sp. nov. (n = 33, PP = 1, BS = 100), and two *Polycera* sp. A from South Africa (PP = 1, BS = 100). *P. quadrilineata* and *P. capitata* (combined with former *P*. *norvegica*) clustered in two distinct and well separated sister clades that formed the sister group to *P. faeroensis*, *P. kernowensis* sp. nov., and *P.* sp. A, which are clustered together in a separate clade, wherein *P. faeroensis* has sister position to *P. kernowensis* sp. nov. The ABGD analysis of the COI data set run with two different models are fully concordant with the clades in the molecular phylogenetic analysis (Fig. 3). Results obtained by PopART showed a network of haplotypes that clearly clustered into four distinct groups coincident with *P. quadrilineata, P. capitata* (combined with former *P*. *norvegica*), *P. faeroensis*, and *P. kernowensis* sp. nov. (Fig. 4). While no correlation was found between the molecular characteristic for each of the four species and the geographical distribution of each of the four species from this complex, *P. capitata* and “*P. norvegica*” recently described from Norway have a widespread distribution in England, Ireland and Norway, and do not show separate clustering. Regarding the supposedly fast-evolving COI marker, uncorrected p-distances within the *P. quadrilineata* clade range 0–3.36%. Whereas minimal uncorrected p-distances between the *P. quadrilineata* clade and *P. capitata* (combined with *P. norvegica), P. faeroensis*, and *P. kernowensis* sp. nov. clades *are* 9.44%, 11.09%, and 10.79% respectively. Uncorrected COI p-distances within the *P. capitata* (combined with *P. norvegica)* clade range from 0.15 to 3.04%. Minimal uncorrected p-distances between the *P. capitata* (combined with *P. norvegica*) clade and *P. faeroensis*, and *P. kernowensis* sp. nov. clades *are* 8.52%, and 8.66% respectively. Uncorrected COI p-distances within the *P. faeroensis* clade are 0.61%; within the *P. kernowensis* sp. nov. clade range 0–2.33%. Uncorrected COI p-distances between *P. faeroensis* and *P. kernowensis* sp. nov. clades range from 5.47 to 6.38% (Table 2).

*Polycera aurantiomarginata* García-Gómez & Bobo, 1984, which was described from Spain and which is also distributed on the West African coasts^22^ belongs to a different *Polycera* clade, considerably different morphologically, and hence is not part of the complex of the European species closely related to *P. quadrilineata.* Therefore *P. aurantiomarginata* is included in the present molecular phylogenetic analysis, but not considered in this study in detail (Fig. 3). An undescribed species (“*Polycera* sp. A”) which was included to a molecular phylogenetic study without any morphological data^14^ is basal to the clade of the European *Polycera* (Fig. 3) but occurs exclusively in South Africa and is out of scope in the present study. For *Polycera marplatensis* Franceschi, 1928^38^ which is partly similar to *P. quadrilineata* molecular data are not available, but this species occurs exclusively in South America and therefore is also out of scope of the present study.

The morphological analysis data were confirmed by molecular phylogenetic results. *P. quadrilineata*, *P. capitata, P. faeroensis*, and *P. kernowensis* sp. nov. are four separate species in the genus *Polycera. P. capitata* and former *P*. *norvegica* are the same species.

**Table 2:** Intragroup (highlighted in bold) and Intergroup genetic distances (%) for COI in the European *Polycera* species complex.

	*P. quadrilineata*	*P. capitata* (including *P*. *norvegica* syn.nov.)	*P. faeroensis*	*P. kernowensis* sp. nov	*P.* sp. A
*P. quadrilineata*	**0–3.36**	9.44–12.68	11.09–13.13	10.79–14.53	10.42–12.5
*P. capitata* (including *P*. *norvegica* syn.nov.)	9.44–12.68	**0.15–3.04**	8,52–9.97	8.66–11.99	9.95–11.21
*P. faeroensis*	11.09–13.13	8.52–9.97	**0.61**	5.47–6.38	8.09
*P. kernowensis* sp. nov	10.79–14.53	8.66–11.99	5.47–6.38	**0–2.33**	8.71–10.42
*P.* sp. A	10.42–12.5	9.95–11.21	8.09	8.71–10.42	**1.4**

### Difficult to distinguish European species within the genus *Polycera*: recognition of the involved species

We obtained a very robust framework of four closely related species from European waters: *Polycera quadrilineata*, *P. capitata, P. faeroensis* and *P. kernowensis* sp. nov. (Figs. 3, 4, for detailed systematic account of all four species see Supplementary information S2). *Polycera quadrilineata* and *P. capitata* are the two common European species and usually present in the shallow marine waters at depths easily accessible for diving (ca*.* 5–40 m), making these species always a focus of attention of various environmental associations, ecological studies and currently also citizen scientists. Therefore it is of high general importance to present a framework for morphological identification of these species complex based on robust molecular data (Tables 1, 2).

Throughout the history of nudibranch studies in Europe, a species similar, but distinct from *P. faeroensis* has been confused with the latter and was never taxonomically recognized^14,39,40^. In the present study this species using robust molecular framework based on a broad geographical sampling and significant external and internal morphological differences (Figs. 1, 2, 9; Table 1) it is for the first time recognized and described as a new species, *Polycera kernowensis* sp. nov. (see details in Supplementary information S2). This species has significantly more rhinophoral lamellae (14–15) than in *P. capitata* (8) and *P. quadrilineata* (11–12) and same is true for the frontal veil appendages (7–8 vs. 4–5) including specimens of similar sizes (Figs. 2, Supplementary information Fig. S1). *Polycera quadrilineata* can reach a similar large size as *P. faeroensis* (commonly the former species is smaller), but the mean number of rhinophoral lamellae in *P. quadrilineata* is significantly smaller (11–12), than in *P. faeroensis* (18–19) (Figs. 2, Fig. S1) and even in largest specimens of *P. quadrilineata* the number of rhinophoral lamellae is smaller than in *P. faeroensis*. In addition, the number of the frontal veil appendages differs with a high support among these three species (Fig. 2, Supplementary information S2, Fig. S1).

**Figure 5:**
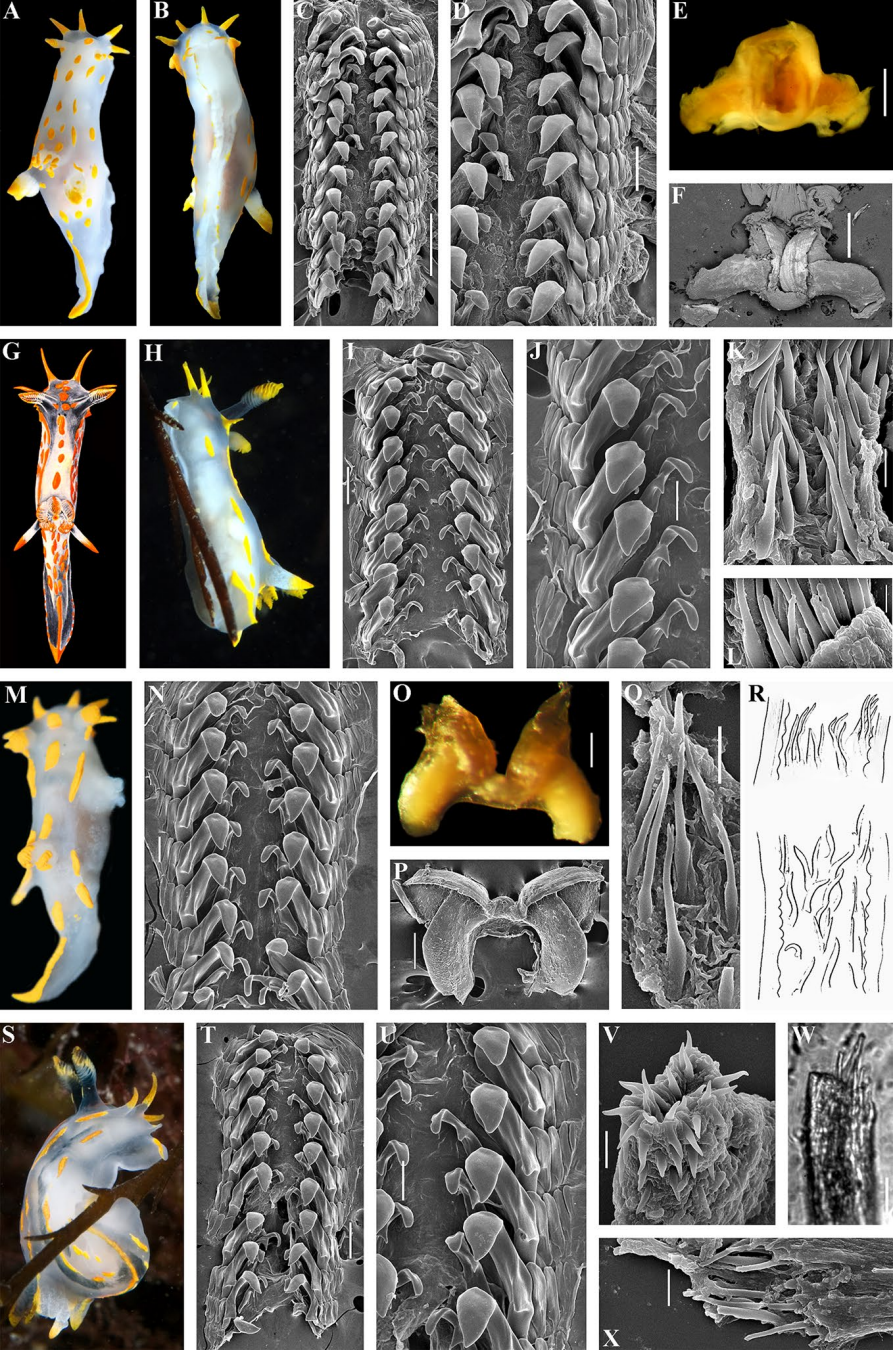
*Polycera quadrilineata* (O.F. Müller, 1776). External and internal morphology, light and scanning electron microscopy and comparison with data from Thompson and Brown^40^ and Bergh^46^. (**A**) Neotype from Norway, Gulen, dorsal view, 25 mm in length, live (ZMMU Op-750). (**B**) Same, ventral view. (**C**) Radula, neotype, scanning electron microscopy (SEM). (**D**) Same, radula details. (**E**) Jaws, neotype, light microscopy. (**F**) Same, SEM. (**G**) Specimen from United Kingdom, Skomer Island, depicted in Thompson and Brown (^40^: pl 18, a). H. Specimen from United Kingdom, Cornwall, Porthkerris, dorsolateral view, live (ZMMU Op-758). (**I**) Radula, SEM (ZMMU Op-758). (**J**) Same, radula details. (**K**) Copulative spines, SEM (ZMMU Op-758). (**L**) Same, details. (**M**) Specimen from Portugal, Setubal, dorsal view, live (ZMMU Op-760). (**N**) Radula, SEM (ZMMU Op-760). (**O**) Jaws, light microscopy (ZMMU Op-760). (**P**) Same, SEM. (**Q**) Copulative spines, SEM (ZMMU Op-760). (**R**) Spines of a specimen from Norway depicted in Bergh (^46^, not in copyright), essentially similar to our present data. (**S**) Specimen from United Kingdom, Cornwall, Porthkerris, dorsolateral view, live (ZMMU Op-756). (**T**) Radula, SEM (ZMMU Op-756). (**U**) Same, radula details. (**V**) Copulative spines from the tip of everted part (shorter than common ones), SEM (ZMMU Op-756). (**W**) Copulative spines (common type), light microscopy (FD 041). (**X**) Same, SEM. Scale bars: c, e, f—500 μm, d, i, j, o, p, t—200 μm, k, q, v, w, x—10 μm, l—5 μm, n, u—100 μm. Photographs: Tatiana Korshunova: (a, b); F.M.F. Driessen (h), (s); Bernard Picton (m). Reproduction of figure from Thompson and Brown^40^ with permission of Gregory Brown, original artist and copyright holder of the images. SEM micrographs and light microscopy photographs: Alexander Martynov.

**Figure 6:**
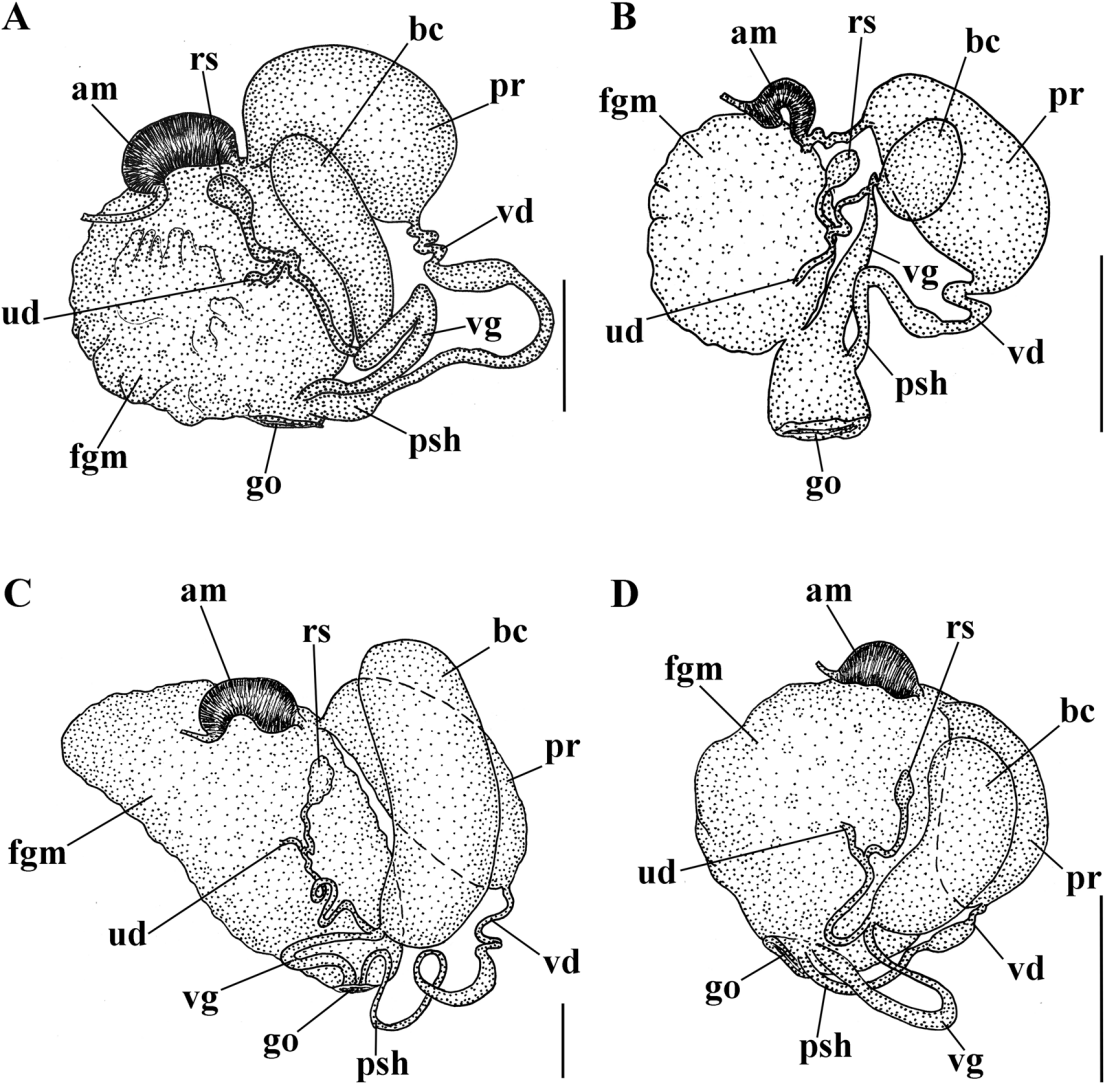
Schemes of the reproductive systems. (**A**) *Polycera quadrilineata*. (**B**) *P. capitata.* (**C**) *P. faeroensis.* (**D**) *P*. *kernowensis* sp. nov. *am* ampulla, *bc* bursa copulatrix, *fgm* female gland mass, *go* genital opening, *pr* prostate, *psh* penial sheath, *rs* receptaculum seminis, *ud* uterine duct, *vd* vas deferens, *vg* vagina. Scale bars: 1 mm.

**Figure 7:**
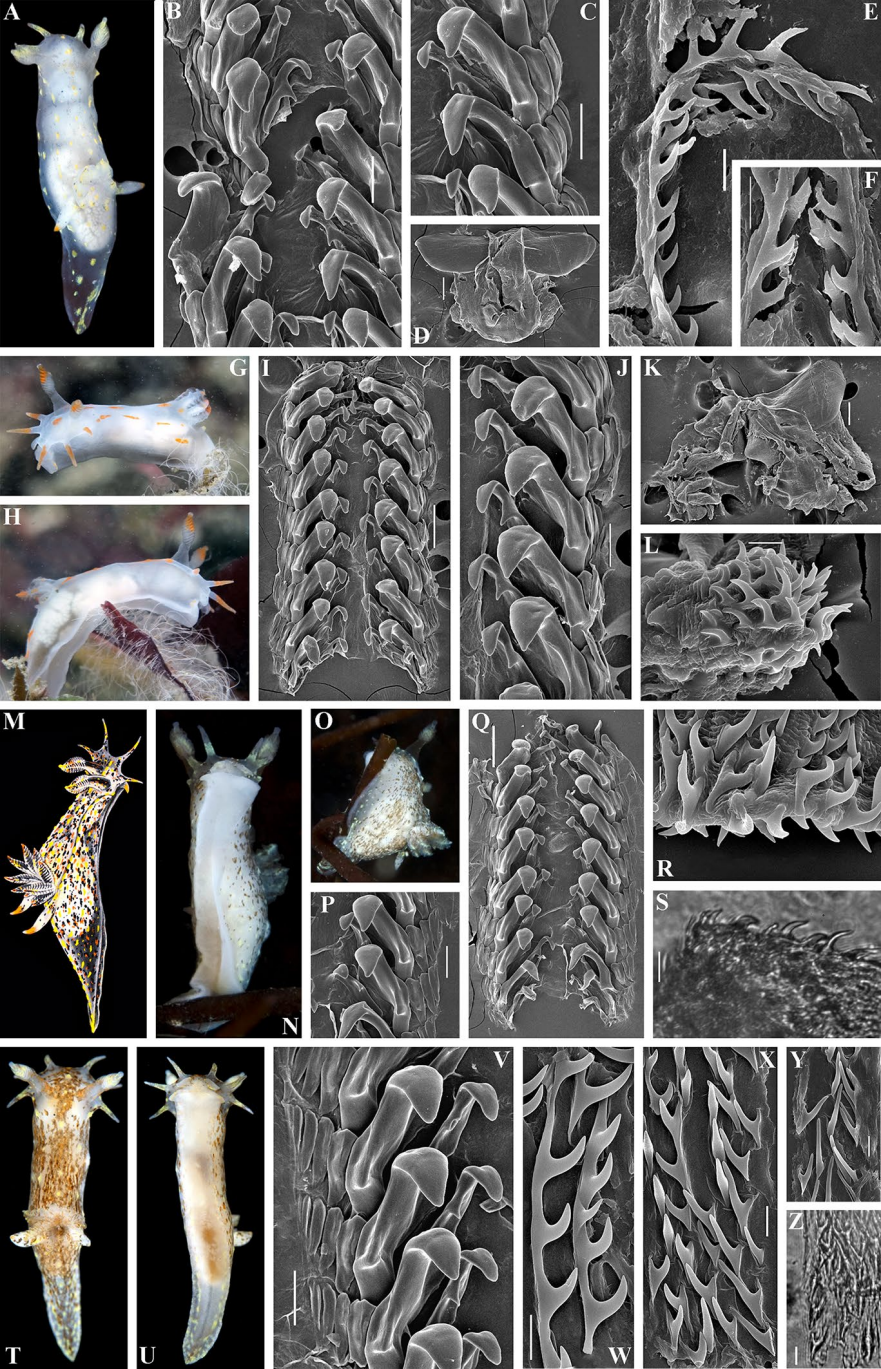
*Polycera capitata* (Alder & Hancock, 1854)^49^. External and internal morphology, light and scanning electron microscopy and comparison with data from Thompson and Brown^40^. (**A**) Specimen from United Kingdom, Cornwall, Porthkerris, dorsal view, live (ZMMU Op-766). (**B**) Radula, SEM (ZMMU Op-766). (**C**) Same, radula details. (**D**) Jaws, SEM. (**E**) Copulative spines, SEM (ZMMU Op-766). (**F**) Same, details. (**G**) Specimen from United Kingdom, Cornwall, Porthkerris, dorsal view, live (ZMMU Op-764). (**H**) Same, lateroventral view. (**I**) Radula, SEM (ZMMU Op-764). (**J**) Same, details. (**K**) Jaws, SEM (ZMMU Op-764). (**L**) Copulative spines, SEM (ZMMU Op-764). (**M**) Specimen from United Kingdom, Lundy Island, depicted in Thompson and Brown (^40^: pl 18, c). (**N**) Specimen from United Kingdom, Cornwall, Porthkerris, ventral view, live (ZMMU Op-765). (**O**) Same, ventral view. (**P**) Radula, details, SEM (ZMMU Op-765). (**Q**) Radula (ZMMU Op-765). (**R**) Copulative spines, SEM (ZMMU Op-765). (**S**) Same, light microscopy. (**T**) Specimen from Ireland, Mullaghmore, Sligo, dorsal view (ZMMU Op-770). (**U**) Same, ventral view. (**V**) Radula, details, SEM (ZMMU Op-770). (**W**–**Y**) Copulative spines, details, SEM (ZMMU Op-770). (**Z**) Same, light microscopy. Scale bars: b–d, j, k, p, v—100 μm, i, q—200 μm, e, f, l, s, w–z—10 μm, r—5 μm. Photographs: F.M.F. Driessen (a), (g), (h), (n), (o); Bernard Picton (t), (u). Reproduction of figure from Thompson and Brown^40^ with permission of Gregory Brown, original artist and copyright holder of the images. SEM micrographs and light microscopy photographs: Alexander Martynov, Tatiana Korshunova.

**Figure 8:**
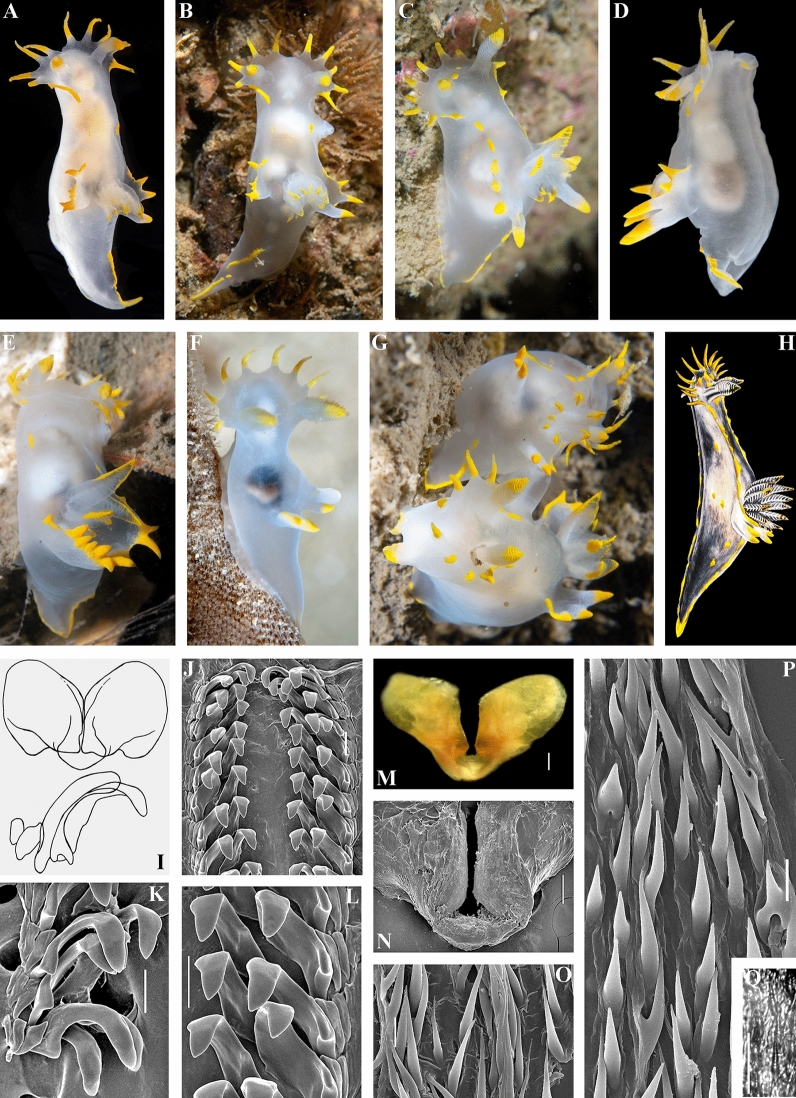
*Polycera faeroensis* Lemche, 1929^41^. External and internal morphology, light and scanning electron microscopy and comparison with data from Thompson and Brown^40^. (**A**, **B**) Specimen from United Kingdom, Belfast Lough, Northern Ireland, dorsal views, live (ZMMU Op-774). (**C**) Specimen from United Kingdom, Strangford Lough, Northern Ireland, dorsolateral view, live. (**D**) Another specimen from Strangford Lough, Northern Ireland, lateral view, live. (**E**) Specimen from the United Kingdom, Skomer Island, Pembrokeshire, dorsal view, live. (**F**) Juvenile specimen from the Strangford Lough, dorsal view, live. (**G**) Copulating specimens from Skomer Island, Pembrokeshire, Wales, dorsal views. (**H**) Specimen from United Kingdom, Isle of Man, depicted in Thompson and Brown (^40^: pl 18, g). (**I**) Schemes of jaws (above) and radula teeth (below) of holotype from the Faeroe Islands. (**J**) Radula, SEM (ZMMU Op-774). (**K**, **L**) Same, details of radula. (**M**) Jaws, light microscopy (ZMMU Op-774). (**N**) Same, details, SEM. (**O**, **P**) Copulative spines, SEM (specimen from Belfast Lough). (**Q**) Same, light microscopy. Scale bars: j–l—100 μm, m, n—200 μm, o–q—10 μm. Photographs: Bernard Picton (a–g). Reproduction of figure from Thompson and Brown^40^ with permission of Gregory Brown, original artist and copyright holder of the images. SEM micrographs and light microscopy photographs: Alexander Martynov.

**Figure 9:**
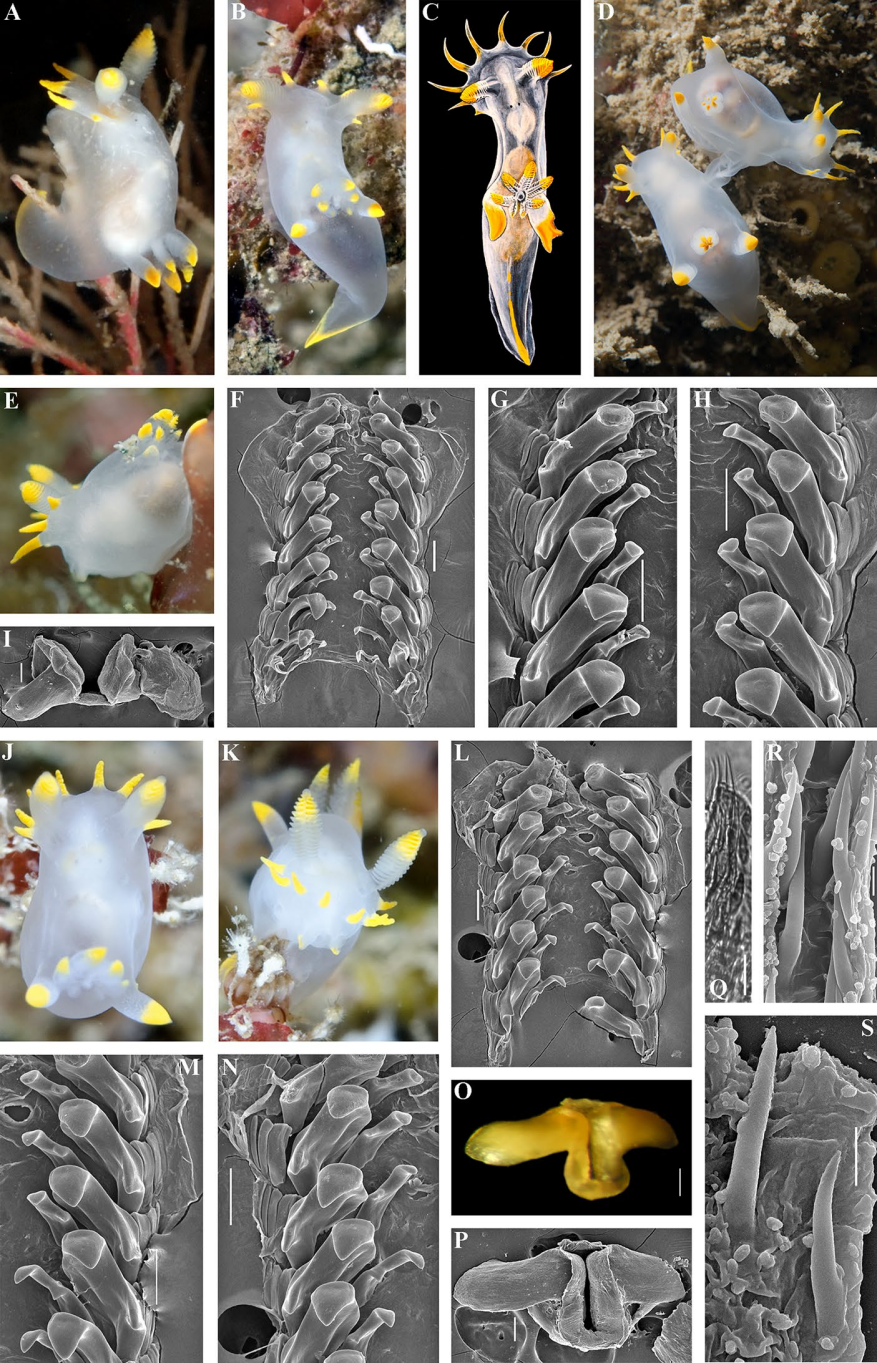
*Polycera kernowensis* sp. nov. External and internal morphology, light and scanning electron microscopy and comparison with data from Thompson and Brown^40^. (**A**) Paratype from United Kingdom, Cornwall, Porthkerris, dorsal view, live (FD 01). (**B**) Paratype from United Kingdom, Cornwall, Porthkerris, dorsal view, live (FD 093). (**C**) Specimen from United Kingdom, Skomer Island, depicted in Thompson and Brown (^40^: pl 18, e) identified as *P. faeroensis* but which has some features that more similar to *P. kernowensis* sp. nov. (**D**) Copulating specimens from the Great Britain, dorsal view, live. (**E**) Paratype from Portugal, Setubal, lateral view, live (ZMMU Op-775). (**F**) Radula, SEM (ZMMU Op-775). (**G**, **H**) Radula details, SEM (ZMMU Op-775). (**I**) Jaws, SEM (ZMMU Op-775). (**J**) Holotype from United Kingdom, Cornwall, Porthkerris, dorsal view, 5.6 mm, live (ZMMU Op-755). (**K**) Same, frontal view, live. (**L**) Radula of holotype, SEM. (**M**, **N**) Radula details (holotype), SEM. (**O**) Jaws (holotype), light microscopy. (**P**) Jaws (holotype), SEM. (**Q**) Copulative spines (holotype), light microscopy. (**R**, **S**) Copulative spines (holotype), SEM. Scale bars: f–i, l–p—100 μm, q—10 μm, r, s—5 μm. Photographs: F.M.F. Driessen (a), (b), (e), (j), (k); (d) Bernard Picton. Reproduction of figure from Thompson and Brown^40^ with permission of Gregory Brown, original artist and copyright holder of the images. SEM micrographs and light microscopy photographs: Alexander Martynov.

**Figure 10:**
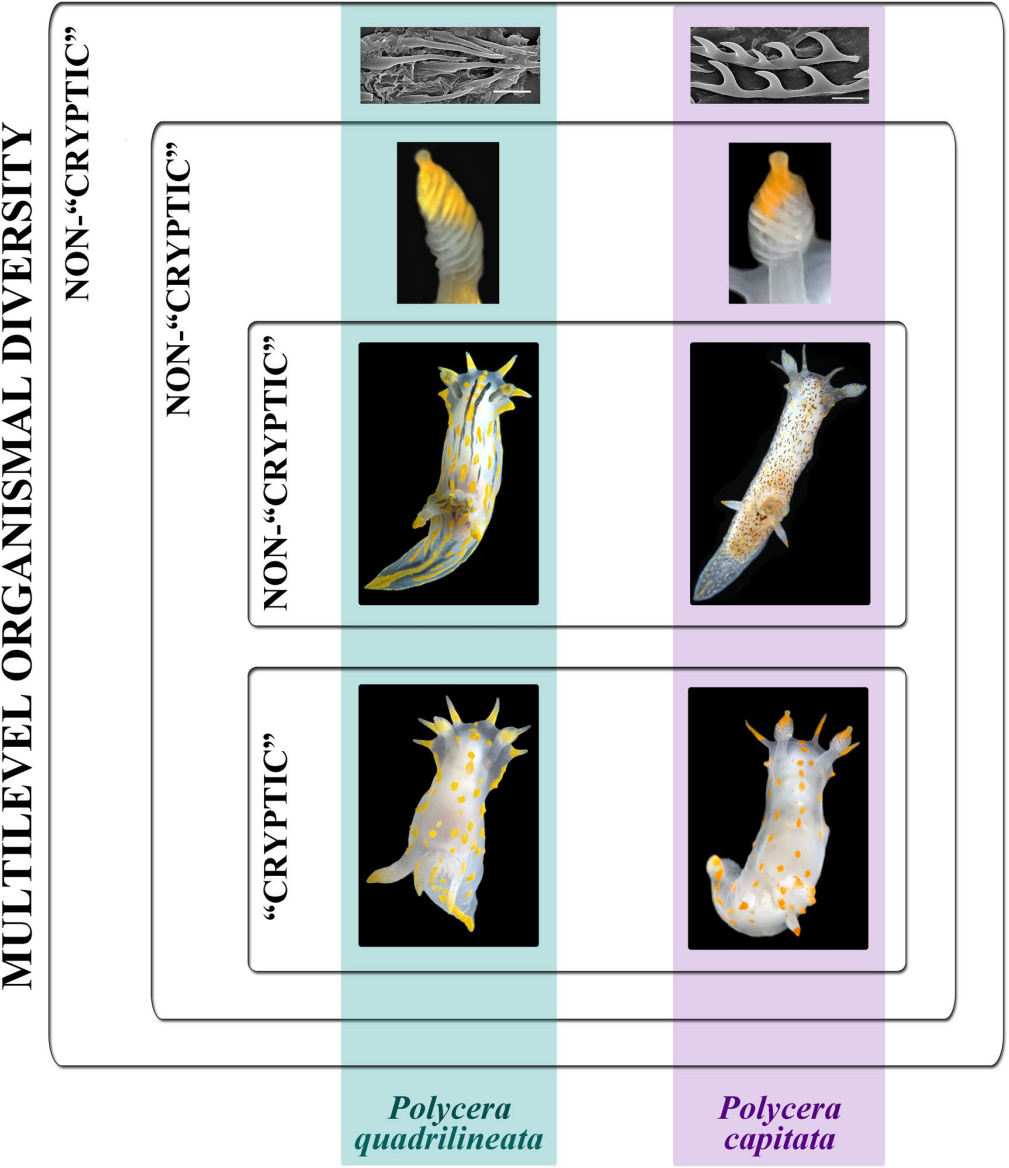
Presentation of the *multilevel organismal diversity* approach using the example of closely related *Polycera quadrilineata* and *P. capitata*: presence of conditional “non-cryptic” and putative “cryptic” components among the same species and provision of fine-scale morphological diagnostic characters even among the “cryptic component” within a single species. Scale bars—10 μm.

Remarkably, in this study we also recovered two closely related species *P. quadrilineata* and *P. capitata* as sister species (Fig. 3), in contrast to a previous study^14^, because taxon selection was previously not exhaustive. True *P. faeroensis* is a more rarely encountered species than *P. quadrilineata* and *P. capitata* (at least in the relatively shallow water environments) and verified molecular data which were aligned with the fine-scale morphological data are presented in this study for the first time (Figs. 1, 2, 3, 4, 5, 6, 7, 8, 9, 10). Previously real *P. faeroensis* were misidentified^14,40^ with its new sister species, here described as *P. kernowensis* sp. nov. (Figs. 8, 9). Our verified data on the morphology of the radula matched well with the original description of *P. faeroensis* from the Faeroe Islands^41^ and a morphological redescription from Sweden^42^.

The results of the statistical test in key diagnostic external features (number of rhinophoral lamellae, frontal veil appendages and gills) in the *Polycera* species complex (Fig. 2, Supplementary information S2), showed that easily accessible external morphological features are distinguishing factors among adult individuals between the species in the complex, with high statistical support (Figs. 2, Supplementary information S2, Fig. S1). Particularly, the number of the rhinophoral lamellae differs with high support among all four species (Figs. 2, 10, Supplementary information S1). It must be noted that differences in rhinophoral lamellae are partly related to the animal length, due to the fact that larger specimens may have larger numbers of rhinophoral lamellae, starting from 0 (i.e. smooth rhinophores) and reaching a mean number of lamellae towards mature stages^43,44^. However, even most closely related sister species *P. quadrilineata* and *P. capitata* were revealed as having smaller, but statistically significant differences in the mean number of the rhinophoral lamellae (mean 8 in *P. capitata* and 11–12 in *P. quadrilineata*) including specimens of similar sizes (Figs. 2, 10, Supplementary information S2).

The significant differences can be further used for the fine-scale diagnostics of these species. This is a very important result for further assessment of the general reliability of the “cryptic species” concept.

## Discussion

### Importance of the true, and not a formal integration of the molecular, morphological, and taxonomic data for applications in ecological and evolutionary studies

The present study is very relevant for the investigation of the general species concept and so-called “cryptic species” concept and also for the currently commonly claimed “integration” of molecular and morphological data. It was already highlighted that the challenge represented by cryptic species has great importance for general biological problems and represents “a window into the paradigm shift of the species concept”^20^. We therefore present in this study the largest currently possible molecular and morphological dataset on a particular nudibranch species complex, which is widely distributed over the vast European region. Molecular data of 178 specimens belonging to four species were involved in the molecular phylogenetic analysis (Fig. 3). Furthermore, for the practitioners, and for researchers who perform environmental monitoring in the European marine waters, it is very important to trustworthily distinguish the four species, including the most difficult to distinguish *P. quadrilineata* and *P. capitata*, in the field and, above all, without complicated, time-consuming and expensive molecular analysis. This is especially important since *P. quadrilineata* complex represents an indicator of biodiversity shifts in relation to climatic changes^27^.

In order to develop a robust framework to make it possible to identify all four species using only easily accessible external characters and applying in the majority of cases, we hereby provide a summary of an exhaustive analysis of molecular and morphological data for all European *Polycera* species (Figs. 1, 2, 3, 4, 5, 6, 7, 8, 9, Tables 1, 2), i.e. every newly available specimen or sequence from European waters belonging to each of the four recognized species (Fig. 3, Tables 1, 2) and not to any other potential “hidden” lineages.

*Polycera quadrilineata* (O. F. Müller, 1776) is one of the oldest species names amongst European nudibranch molluscs^45,46^. Since then there were numerous attempts to separate more species similar to *P. quadrilineata* (see the Supplementary information S1 for details on the systematics of *P. quadrilineata*), however all of them without a detailed morphological analysis were later considered to be synonyms of *P. quadrilineata*^40^. Using molecular data, Driessen et al.^21^ showed two lineages among European “*P. quadrilineata*” for the first time. Recently one of these lineages has been formally described as *Polycera norvegica*^14^. The description proposed the species to occur exclusively in Norway. According to our data, applying a very broad geographic sampling in frames of the present study (see e.g., Figs. 3, 4) the recently named “*Polycera norvegica*” definitely is not distributed solely in Norway, but is a species widely distributed in other European countries, such as the United Kingdom and Ireland (Supplementary information, Table S1). Furthermore, in that publication, the numerous synonyms of *P. quadrilineata* were neither listed nor investigated, and its synonymy was just referred to the internet database MolluscaBase^47^.

In the present study we carefully investigated all existing available synonyms of *P. quadrilineata* (Figs. 1, 2, 3, 4, 5, 6, 7, Table 1, Supplementary information Fig. S1). Therefore, being part of the difficult to distinguish *P. quadrilineata* complex, for which more than ten synonyms have been suggested, and having a broad distribution in Europe, “*P. norvegica*” could have already been described. If “*P. norvegica*” had no clear recognizable chromatic variants which immediately distinguished it from *P. quadrilineata* it would be difficult to assess a potential synonym. However, “darker” chromatic variants (Fig. 1IV–VII) are clearly different between *P. quadrilineata* and “*P. norvegica*”, which makes a solid base for the search for such previously described variants within taxonomic synonyms. During this study we identified two available taxonomic names that contain chromatic variants with darker colouration without evident stripes, and those can be thus excluded from the striped variants of true *P. quadrilineata* (Figs. 1, 2, 5, 7). These available names are *Polycera ornata* d'Orbigny, 1837 and *Thecacera capitata* Alder & Hancock, 1854. For *Polycera ornata* a main morph with red–orange lines was described^48^ and in addition a morph with weaker orange and black spots was mentioned. Because of a main morph with orange-reddish lines in *Polycera ornata*, and having only an old painting published, it is difficult to surely distinguish the morph from *P. quadrilineata*. Furthermore, for *P. ornata* a “tiger-like” colouration was indicated for the darker morph^48^, that may imply stripes, as in true *P. quadrilineata*. Thus, to assign *Polycera ornata* to this species would be ambiguous. Also, the type material of *Polycera ornata* was not saved.

Instead, for *Thecacera capitata* in the original description by Alder & Hancock (1854) the colouration was solely indicated as “freckled with brownish greenish”^49^ that immediately allows exclusion of any morphs with evident black stripes, that are present only in true *P. quadrilineata* (Figs. 1, 5). *Thecacera capitata* was later confirmed as belonging to the genus *Polycera* and not to *Thecacera*, including study of the type material^40^; presence of rhinophoral sheaths for *P. capitata* was thus indicated mistakenly^49^. *Polycera capitata* was partly redescribed with aid of the type material regarding external features and the external colouration^40^ and exactly matched the chromatic variant V in *P. norvegica* but not any chromatic variants of *P. quadrilineata* (Figs. 1, 7M). In this study we additionally studied the saved radula from the type specimen of *Polycera capitata* as was figured in the original description^49^. The radula has only four outer lateral teeth and thus fits well to the most rigorous assessment of the radular characters in this species, including “*P. norvegica*”^14^.

This combination of the external and internal data excludes the possibility that *Polycera capitata* is a synonym of *P. quadrilineata* but instead conforms closely to the characters that were recently described for *P. norvegica*^14^. We have involved numerous specimens from the United Kingdom and Ireland—that matched well morphologically to the original description of *P. capitata* (Figs. 1, 7)—in the present molecular analysis. The present study shows that darker chromatic variants V–VII without distinct stripes are characteristic solely for *P. capitata* (Figs. 1, 7, 10). Furthermore, these darker chromatic variants of *P. capitata* are very common throughout the United Kingdom from Cornwall in the south to the Scotland in the north, including the type locality at St. Ives, Cornwall according to the available photographic data^50,51^. These data were already available, but an essential external similarity of “*P. norvegica*” to *P. capitata* was missed and it was incorrectly stated that the species is restricted to Norway^14^. “*P. norvegica*” syn. nov. thus becomes a junior synonym of *Polycera capitata.* Additionally, (with permission) we present a copy of plate 18c from Thompson & Brown (1984)^40^ where the external features illustrated for *Polycera capitata* correspond exactly with our morphological data for *P. capitata* (Fig. 7M). Therefore, our present taxonomic assignment of *P. capitata* is robustly supported by the abundant morphological (including statistical study), molecular and distributional data (Figs. 1, 2, 3, 4, 5, 6, 7, Table 1, Supplementary information, Table S1, Fig. S1). In the present study we therefore restore the species *P. capitata* and *P. norvegica* becomes its junior synonym.

Importantly, what appears to be a particular taxonomic problem, has in reality general importance for an array of biological fields since it clearly shows that without true integration of an “old” taxonomic knowledge, that tends to be neglected currently, with modern morphological and molecular data, an appropriate study of world biodiversity cannot be performed. The “cryptic species problem” therefore does not emerge only recently, but was always part of taxonomy since the Linnean era. A formal integration of the molecular and morphological data and inaccurate claim for “cryptic species” led to omission of a synonym for a common, species which is important for ecological monitoring. Therefore, the importance of true and not just a formal integration of molecular, morphological and taxonomic data are here specially highlighted and a practical set of methods is proposed below.

### Periodic-like framework for the recognition diagnosable characters in the species complexes

Periodic-like as well as parallel-like patterns in biological applications have been discussed for a long time and successfully applied for protein structure^52^, but they are still not widely used practical tools for taxonomy and phylogeny. Several recent studies on different groups, such as rodents^53^ and fishes^54,55^ robustly confirmed the existence of periodic patterns during development of morphological characters at a genomic level, particularly concerning chromatic variants and thus are directly connected with the present *Polycera* case. Notably, the ontogenetic periodicity is based on periodicity of homeobox and other developmental gene systems in animals and plants^56,57^, thus approaching chemical periodicity. Recently an evident periodicity was revealed for a higher-level organism group using an ontogenetic phylotypic periods/stages approach, that consistently links the genomic and morphological levels^44^. There were attempts to describe chromatic variants within another nudibranch family Chromodorididae under the term “colour groups”^58^ or as different colour morphs in frames of a phylogeny^59,60^, but not in a periodic framework. However, when similar morphs of different closely related species are mapped in the same horizontal sections, the partial periodicity can be clearly revealed^18^.

During individual development, this was investigated for *Polycera quadrilineata*^36^, the darker colouration appeared during later stages of the ontogeny, and similar patterns of the ontogeny of the sister species *P. capitata* must present in parallel, which makes the biological grounds for the periodic-like approach. Such an approach is a practical one and helps to reveal fine distinguishing details among apparently very similar morphs (e.g. chromatic variants) and also potentially not yet discovered morphs of the closely related species. Currently we have no information about which particular genes underlie any common genetic basis in the polychromatic nudibranchs^61^, but it must inevitably imply similar developmental genes basis and it was recently used for delineation of a very difficult nudibranch species complex of the genus *Amphorina*^18^. A North Pacific species, *Polycera atra* MacFarland, 1905, provides evidence for the existence of the underlying similar genomic basis, that appears in parallel in various phylogenetic lineages. According to the present analysis it represents a taxon which is only distantly related to the *P. quadrilineata* complex (Fig. 3), and potentially may belong to a separate genus (a general revision of the family Polyceridae is pending), but nevertheless exhibits similar chromatic variants^62^. As in the *P. quadrilineata* complex, about eight chromatic variants of *P. atra* are recognized. These are used in the present study to align the chromatic variants found within the European *Polycera* species (Fig. 1). Such parallel appearance of the similar chromatic variations in relatively distantly related clades is well matched to the parallel appearance of the shell morphs within gastropod molluscs^63^, for which a similar genomic base has already been confirmed^64^.

The present *Polycera* chromatic polymorphism is a clear case of periodic appearance of similar colour morphs among phylogenetically related but different species which has robust support from a large molecular dataset (Figs. 1, 3). *Polycera quadrilineata* also possesses stripe patterns in their colouration (Fig. 1V–VIII), and similar patterns were recently showed as underlain by ontogenetic periodicity in various groups^54,55^. Within *P. capitata* instead darker forms (Fig. 1V–VIII) appear in parallel, without distinct stripes, but with remnants of a faint stripe-like pattern (Fig. 1VI). In both *P. faeroensis* and *P. kernowensis* sp. nov. the chromatic variants IV–VII are not yet discovered (Fig. 1). This makes the method a practical tool for revealing a diagnosable character within putative “cryptic species complexes”. While biologists from non-taxonomic fields or experienced practitioners have the task to identify *Polycera* in the field, they will be guided by a periodic-like mapping (Fig. 1) which is based on accurate taxonomy and robust molecular phylogenetic data (Figs. 3, 4). It will be easier to exclude these variants that do not occur (as striped or heavily spotted variants are not yet found in *P. faeroensis* and *P. kernowensis* sp. nov.), and instead carefully investigate similar chromatic variants II and III in two sister species *P. quadrilineata* and *P. capitata* (Fig. 1).

When such periodic-like mapping becomes a routine part of biodiversity studies, the accumulated data will allow the presentation of small, fine-scale distinguishing characters even between such highly similar orange-spotted chromatic variants that are present in both *P. quadrilineata* and *P. capitata*. At present, they remain difficult to distinguish, but using available data we can preliminarily conclude that in *P. capitata* orange spots are more commonly smaller and more rarely form lines, than in *P. quadrilineata* (Figs. 1, 2, 5, 7). A statistical test of the diagnostic value of the external characters, that were mapped in the periodic-like framework, revealed that number of the rhinophoral lamellae, though it may overlap in juvenile specimens, in adult specimens is different with a statistical high support including among these difficult to distinguish chromatic variants II and III between *P. capitata* and *P. quadrilineata* (Fig. 2, Supplementary information S1, Fig. S1). Thus, the number of the rhinophoral lamellae (a very easy to check character even on the photographs), can be used as an additional verification in case of similar chromatic variants II and III between *P. capitata* (commonly less than ten rhinophoral lamellae in adults) and *P. quadrilineata* (commonly more than ten rhinophoral lamellae in adults)*.*

The periodic-like mapping of the chromatic variants revealed that orange dots on the body sometimes occur, as well as potentially darker morphs in true *P. faeroensis* (Fig. 1II–VIII), but patterns and quantity differ between *P. quadrilineata* and *P. capitata* (Fig. 1II–VIII). Previously all four species of the European *Polycera* complex have been confused with each other^40,65^ also because these chromatic variants were previously never accurately mapped with each other, but instead it was commonly noted that similar to *P. quadrilineata* and *P. capitata* yellow–orange dots were sometimes present on the body in *P. faeroensis*, which immediately misled practitioners that have had tasks to identify animals in the field. Further, this profound confusion among identification of European *Polycera* persists even using modern molecular tools as *P. faeroensis* until the present study was not distinguished from *P*. *kernowensis* sp. nov., despite the presence of robust external, internal, and molecular differences (Figs. 1, 2, 3, 4, 8, 9). Thus, the periodic-like mapping of the polychromatic variants in combination with statistical analysis of the diagnostic characters is an important tool in addition to the molecular phylogenetic analysis. It will be particularly important since *P. quadrilineata* and *P. capitata* are two of the most common nudibranch species in Europe, and particularly in the UK the further testing of their chromatic variance already attracted attention^51^ and further attraction of various environmental and educational organizations and citizen scientists is expected. Our present study builds a major framework for further broader testing of the colour polymorphism and other distinguishing features in the *Polycera* species complex that will have importance for the development of documenting the fine-scale diversity not only in other nudibranchs, but in the variety of multicellular organisms.

### Practical guidelines how to perform a taxonomic study in the molecular era

The present study evidently shows that “cryptic” and “non-cryptic” components are present within the same species. This further significantly undermines the “cryptic species” concept, which recently was already questioned^4,10–12^. The presence of both “cryptic” and “non-cryptic” components is demonstrated here for sister species *P. quadrilineata* and *P. capitata* sharing externally similar chromatic variants II and III (Fig. 1), which however can be distinguished by the morphological features of another level (Figs. 5, 7). Particularly, the internal features of the shape and size of copulative spines allow in 100% cases to confirm the identity of both species (compare Figs. 5K, L, Q, R, V–X and 7E, F, L, R, S, W–Z), even without aid of molecular data this undermines the proposal that these species are morphologically “cryptic”. In our recent study^12^ we already showed how the underestimation of the taxonomic and morphological data resulted in a long-term omission of reliable multilevel differences in the nudibranch *Trinchesia* species complex. Recently, there are proposals that taxonomy should be an integrative study^66–68^. However, in current common practice “integration” unfortunately mainly means performing a molecular study on some selection of specimens and then morphological features listed “in addition”, rather as an auxiliary information. The best confirmation of this is the recent *Polycera* study^14^, when crucial previous taxonomic information was omitted, and while some morphological diagnostic features were revealed, both their stability and variability (and hence, their usefulness for taxonomic diagnostics) were over- or underestimated that subsequently led to an incorrect statement about “general crypticity” of the involved species.

To avoid this, practical guidelines proposing how to perform a taxonomic study in the molecular era are outlined here: (1) make a selection of a taxonomic group and appropriate specimens; (2) make a relevant morphological study in a given group, including for example scanning electron microscopy of previously commonly used diagnostic characters in the given group; (3) ensure that ontogenetic information is considered during taxonomic assessment, because adult diagnostic characters can be considerably transformed at different ontogenetic stages, whereas adult paedomorphic characters can be easily misidentified with juvenile transitive features^44,69–71^; (4) make an appropriate bibliographic study, to exhaustively study the synonymy of a studied taxa/species group, importantly, not just as a reference to a taxonomic data base, but to perform a real study of original sources; (5) molecular study selected and taxonomically checked specimens with commonly used genetic markers in a given group; (6) compare the results of the morphological (step 2) and taxonomic investigation (steps 3, 4) with the molecular (step 5) results; (7) in case of finding discrepancies between previously commonly assessed diagnostic characters in a given species group and results of molecular phylogeny, respective diagnostic features of specimens in question should be presented in a periodic-like framework of the parallel rows, that will enable their detailed comparison and further search for fine-scale differences between given rows for each of the closely related species; (8) in case difficult to distinguish variants are present among the same parallel rows, a statistic study of the relevant diagnostic features should be performed in order to reveal fine-scale differences among closely related species; (9) a complete study at a given time and using current research possibilities should result in fine-scale taxonomic diagnoses for all closely related species in a given taxa/complex (including new taxa); (10) test the established framework by further investigation of a given group with new materials and data.

Applying the above described methodology and using a large dataset we were able to exhaustively investigate the European *Polycera* species complex (Figs. 1, 2, 3, 4) which was recently specially termed as a “cryptic one”^14^. As a result, we definitely assessed every particular specimen (or sequence data) to a particular species: *P. quadrilineata*, *P. capitata*, *P. faeroensis,* and *P.kernowensis* sp. nov. (Figs. 1, 3, 5, 9). For each of these species we provided diagnostic characters that are based not just on traditional taxonomic “listing” of some features, but on statistically supported analysis of key external features. Particularly, in addition to the molecular phylogenetic study and periodic-like mapping of the chromatic variance we tested the most commonly used diagnostic characters within the *Polycera* species complex, such as number of the rhinophoral lamellae, number of frontal veil appendages and number of gills using statistical tests (Fig. 2). As a result, we discovered that the four species differ in number of rhinophoral lamellae with high statistical support (Fig. 2, Supplementary information S2). With this study, we integrated molecular and morphological data and moreover recovered the multilevel diagnosable diversity within the given species complex, including presenting the distinguishing characteristics between closely related and most difficult to distinguish species from this complex: *P. quadrilineata* and *P. capitata*. When specimens of these species belong to similar chromatic variants II and III, it will be possible to diagnose it using not only clearly different micromorphological features of copulative spines, but also easily accessible external morphological features (Figs. 2, 5, 7, Supplementary information, Fig. S1). They possess consistent molecular and morphological units, and importantly, there are no signs for further hidden molecular lineage among European *Polycera* species.

### Multilevel organismal diversity approach and conclusions on “cryptic species” problem

According to the suggested approach, when knowing more difficult to distinguish species within a species-complex in other organism groups^8,12,72^, putative crypticity means that characteristics were not investigated at a maximally possible fine-scale level (despite considerable efforts) using fine morphology-based comparative rows of parallel characteristics. Without comparative rows of parallel characters it is especially difficult to distinguish species with a highly simplified morphology, for example tiny paedomorphic, decelerated burrowing polychaete worms^13,73^. Using a lineage-based approach, as shown recently^1^, is not enough for species definition. Every new specimen, even a clone must have either smaller or larger differences from any other specimens^12^. It is therefore biologically impossible that a species can be morphologically “completely cryptic”. Conclusions about a “cryptic species” appeared when fine-scale differences (either macro- or micromorphological) were not detected at a present level of knowledge in given groups. It was already concluded most recently that in several cases, potential diagnostic characters were hidden within broadly overestimated “intraspecific variations”^14^ and that a search for the fine-scale morphological differences using various modern methods is important part of future research, because otherwise, for example in palaeontology, species diagnostics will be greatly compromised^74^.

The present data evidently imply that both *P. quadrilineata* and *P. capitata* species comprise putative “cryptic” and conditional “non-cryptic” components. We generated a scheme to clearly show this phenomenon (Fig. 10). *P. quadrilineata* and *P. capitata* have similar chromatic variants (Fig. 1II–III, 10) at a “macromorphological” level. Therefore they conditionally represent a “cryptic component” but are readily different by other chromatic variants (IV–VIII). This additionally has a strong statistical support in other external macromorphological features, such as the number of rhinophoral lamellae, and therefore manifesting a “non-cryptic component” for the diversity within *Polycera* (Figs. 2, 10). Subsequently, at a “micromorphological” level, the differences in shape and size of the copulative spines allow clear and unambiguous distinction of *P. quadrilineata* and *P. capitata* by “non-cryptic components” (Fig. 10). Further, at a molecular level all four *Polycera* species demonstrate significant genetic distances (Figs. 3, 4; Table 2).

Particularly, the special emphasising about “crypticity” of *P. capitata* in Norway^14^ did not help to correctly recognize taxonomic and distributional data for this species, but instead led to important omission that in reality *P. capitata* is not restricted to Norway, but common throughout waters of the middle and Northern Europe (Figs. 3, 4; Supplementary information Table S1). By results of the present study, we can further extend and strengthen our recent arguments^4,12^ and conclude that it is highly counterproductive to divide the biological diversity into “cryptic”^8,14,75^ and “non-cryptic” components since both may be presented within a single species. Additionally, the term “cryptic species” can be misleading because of its continued usage in recent publications, but in a completely different ecological meaning of a well camouflaged species^76,77^.

We propose that the central biological phenomenon of a species that currently attempted to be disrupted into “cryptic” and “non-cryptic” ones can be instead universally designated as *multilevel organismal diversity* (Fig. 10). This term will avoid artificial division of the very complex biological phenomenon of diversity. *Multilevel organismal diversity* encompasses numerous patterns and processes of various levels, from which most notable and readily recognizable are morphological and molecular levels, each with numerous intersected sublevels, ultimately forming complex functional living organisms.

## Supplementary Information

Supplementary Information.

## Data Availability

Sequences will be deposited in GenBank, and can be consulted accessing GenBank, with sequence IDs listed in Table S1.
