# Supplementary material for: The multilevel organismal diversity approach deciphers difficult to distinguish nudibranch species complex
Source: Sci Rep. 2021 Sep 15;11:18323. doi: 10.1038/s41598-021-94863-5 (PMC8443629; doi:10.1038/s41598-021-94863-5)
Supplement: Supplementary file 1 — Supplementary Information. [file 41598_2021_94863_MOESM1_ESM.pdf]

Supplementary Information S1. Protocols of the DNA Extraction and Polymerase Chain Reaction (PCR).

Protocol 1.

For molecular analysis a small pieces were used for DNA extraction with Syntol S-Sorb kit by Syntol Company, according to the producer's protocols. Extracted DNA was used as a template for the amplification of partial sequences of the COI, and 16S. The primers that were used for amplification are LCO 1490 [GGTCAACAAATCATAAAGATATTGG (Folmer *et al.* 1994)];
HCO 2198 [TAAACTTCAGGGTGACCAAAAAATCA (Folmer *et al.* 1994)];
16S arL [CGCCTGTTTAACAAAAACAT (Palumbi *et al.* 2002)];
16S R [CCGRTYTGAACTCAGCTCACG (Puslednik & Serb 2008)]. Polymerase chain reaction (PCR) amplifications were carried out in a 20-μL reaction volume, which included 4 μL of 5x Screen Mix (Eurogen Lab), 0.5 μL of each primer (10 μM stock), 1 μL of genomic DNA, and 14 μL of sterile water. The amplification of COI was performed with an initial denaturation for 1 min at 95° C, followed by 35 cycles of 15 sec at 95° C (denaturation), 15 sec at 45° C (annealing temperature), and 30 sec at 72° C, with a final extension of 7 min at 72° C. The 16S amplification began with an initial denaturation for 1 min at 95° C, followed by 40 cycles of 15 sec at 95° C (denaturation), 15 sec at 52° C (annealing temperature), and 30 sec at 72° C, with a final extension of 7 min at 72° C. Sequencing for both strands proceeded with the ABI PRISM® BigDye™ Terminator v. 3.1. Sequencing reactions were analysed using an Applied Biosystems 3730 DNA Analyzer. COI sequences were translated into amino acids for confirmation of the alignment.

Protocol 2.

For molecular analysis a small pieces were used for DNA extraction with GenElute® Mammalian Genomic DNA kit by Sigma Laboratories, Inc., according to the producer's protocols. Extracted DNA was used as a template for the amplification of partial sequences of the COI, and 16S. The primers that were used for amplification are
LCO 1490 [GGTCAACAAATCATAAAGATATTGG (Folmer *et al.* 1994)];
HCO 2198 [TAAACTTCAGGGTGACCAAAAAATCA (Folmer *et al.* 1994)];
16S arL [CGCCTGTTTATCAAAAACAT (Palumbi *et al.*,1991)];
16S R [CCGGTCTGAACTCAGATCACGT (Palumbi *et al.*,1991)]. Polymerase chain reaction (PCR) amplifications were carried out in a 50-μL Reaction Mix volume, which included
36,75 μl of PCR water, 5 μl of 10xPCR buffer, 5 μl of dNTP's (2,5 mM), 0,50 μl of each primer (50 μM), 1 μl of BSA (20 mg/ml), 1 unit of BiothermPlus Taq and 1 μl of template DNA.
The amplification of COI was performed with an initial denaturation for 5 min at 94° C, followed by 38 cycles of 30 sec at 94° C, 45 sec at 40°C, and 45 sec at 72° C, with a final extension of 7 min at 72° C, and terminated by cooling to 4° C for 5 minutes. The 16S amplification began with an initial denaturation for 2 min at 95° C, followed by 37 cycles of 40 sec at 95° C, 40 sec at 50° C, and 60 sec at 72° C, with a final extension of 5 min at 72° C, and terminated by cooling to 4°C for 5 minutes. Sequencing reactions were analysed using https://www.baseclear.com analyzer. COI sequences were translated into amino acids for confirmation of the alignment.

References

Folmer, O., Black, M., Hoeh, W., Lutz, R. & Vrijenhoek, R. (1994). DNA primers for amplification of mitochondrial cytochrome c oxidase subunit I from diverse metazoan invertebrates. Molecular Marine Biology and Biotechnology, 3, 294–299.

Palumbi, S.R., Martin, A.P., Romano, S.L., McMillan, W.O.D., Stice, L. and Grabowski, G., (1991). The Simple Fool's Guide to PCR. Department of Zoology, University of Hawaii, Honolulu.

Palumbi, S. R., Martin, A. P., Romano, S., McMillan, W. O., Stice, L. & Grabowski, G. (2002). The simple fool's guide to PCR. Honolulu: University of Hawaii.

Puslednik, L. & Serb, J. M. (2008). Molecular phylogenetics of the Pectinidae (Mollusca: Bivalvia) and effect of increased taxon sampling and outgroup selection on tree topology. Molecular Phylogenetic and Evolution, 48, 1178–1188.

Supplementary Information S2. Material examined and Systematics.

Phylum Mollusca
Order Nudibranchia Cuvier, 1817
Family Polyceridae Alder & Hancock, 1845

Genus *Polycera* Cuvier, 1816
Type species *Doris quadrilineata* O. F. Müller, 1776

Polycera quadrilineata **(O.F. Müller, 1776)**

(Figures 1–5, 6A, 10, S1; Tables 1, 2, S1, S2)

Synonymy:
Doris quadrilineata Müller, 1776: 229, 1779: 37, figs 4–6.
Doris flava Montagu, 1804: 79, pl. 7, fig. 6.
Doris cornuta Rathke, 1806: 29, pl. 45, figs 1–3.
Polycera lineatus Risso, 1826: 30.
Polycera ornata d'Orbigny, 1837: 9–12, pl. 107.
Polycera quadrilineata var. *nonlineata* W. Thompson, 1840: 92, pl. 2, fig. 6.
Polycera typica W. Thompson, 1840: 92–93, pl. 2, fig. 5.
Polycera varians M. Sars, 1840: 159.
Polycera quadrilineata var. *mediterranea* Bergh, 1879: 610–613, pl. 9, fig. 10–11, pl. 10, fig. 8–12.
Polycera quadrilineata var. *nigropicta* Ihering, 1886: 47, pl.1, fig. 5.
Polycera quadrilineata var. *nigrolineata* Dautzenberg & Durouchoux, 1913: 8.
Polycera salamandra Labbé, 1931: 22–24, fig. 2.
Polycera quadrilineata – Bergh, 1879: 602–610, pl. IX. figs 1–11, pl 10, figs 1–12, pl. 11, fig. 1.; Ihering, 1886, pl. 2, fig. 7.
Non *Polycera quadrilineata* sensu Thompson & Brown, 1984 and auctorum (mixture of several species).

Type locality: Norway.

Material examined. Neotype. NE Atlantic, Norway, Gulen Dive Center, Norway (60° 57'27.11" N 5° 07' 47.10" E), 5–15 m depth, stones, coll. T.A. Korshunova, A.V. Martynov, 16.03.2014, (ZMMU Op-750, 25 mm in length, live). Other materials. NE Atlantic, Norway, Gulen Dive Center, Norway (60° 57'27.11" N 5° 07' 47.10" E), 5–15 m depth, stones, coll. T.A. Korshunova, A.V. Martynov, 16.03.2014, (ZMMU Op-750, 25 mm in length, live). NE Atlantic, Porthkerris, United Kingdom (50° 03,57' N 05°03,55' W), 8 m depth, 24.09.2013, coll. F.M.F. Driessen R. Dekker, M. Spierenburg, (FD 005, 6.5 mm in length, live). NE Atlantic, North Sea, Eastern Scheldt, Netherlands (51° 37,75' N 03°54,82' E), 2–12 m depth, 30.10.2011, coll. F.M.F. Driessen, (FD 014, 17.1 mm in length, live).NE Atlantic, Porthkerris, United Kingdom (50° 03,57 N 05°03,55' W), 8 m depth, 24.09.2013, coll. F.M.F. Driessen R. Dekker, M. Spierenburg, (FD 019, 7.7 mm in length, live). NE Atlantic, North Sea, Eastern Scheldt, Netherlands (51° 37,75' N 03°54,82' E), 2–12 m depth, 30.10.2011, coll. F.M.F. Driessen, (FD 027, 18.0 mm in length, live). NE Atlantic, Porthkerris, United Kingdom (50° 03,57' N 05°03,55' W), ca. 8 m depth, 24.09.2013, coll. F.M.F. Driessen R. Dekker, M. Spierenburg, (FD 030, ca. 8.1 mm in length, live). NE Atlantic, Porthkerris, United Kingdom (50° 03,57' N 05°03,55' W), 8 m depth, 24.09.2013, coll. F.M.F. Driessen R. Dekker, M. Spierenburg, (FD 038, 7.3 mm in length, live).

NE Atlantic, Porthkerris, United Kingdom (50° 03,57' N 05°03,55' W), 8 m depth, 24.09.2013, coll. F.M.F. Driessen, R. Dekker, M. Spierenburg, (ZMMU Op-756, 8.9 mm in length, live). NE Atlantic, North Sea, Eastern Scheldt, Netherlands (51° 37,75' N 03°54,82' E), 2–12 m depth, 30.10.2011, coll. F.M.F. Driessen, (FD 040, 17.4 mm in length, live). NE Atlantic, North Sea, Eastern Scheldt, Netherlands (51° 37,75' N 03°54,82' E), 2–12 m depth, 30.10.2011, coll. F.M.F. Driessen, (FD 044, 16.2 mm in length, live). NE Atlantic, North Sea, Eastern Scheldt, Netherlands (51° 37,75' N 03°54,82' E), 2–12 m depth, 30.10.2011, coll. F.M.F. Driessen, (FD 045, 16.3 mm in length, live). NE Atlantic, North Sea, Eastern Scheldt, Netherlands (51° 37,75' N 03°54,82' E), unknown depth, 29.06.2013, coll. F.M.F. Driessen, (FD 048, 11.2 mm in length, live).NE Atlantic, Porthkerris, United Kingdom (50° 03,57' N 05°03,55' W), 12 m depth, 25.09.2013, coll. F.M.F. Driessen (ZMMU Op-757, 6.7 mm in length, live). NE Atlantic, Porthkerris, United Kingdom (50° 03,57' N 05°03,55' W), 8 m depth, 24.09.2013, coll. F.M.F. Driessen R. Dekker, M. Spierenburg, (FD 054, 6.1 mm in length, live). NE Atlantic, North Sea, Sylt, Germany (54° 25,87' N 08°10,40' E), unknown depth, 15.10.2012, coll. Arjan Gittenberger, Niels Schrieken (FD 055, 7.2 mm in length, live). NE Atlantic, North Sea, Skagerrak, Location 15, Sweden (58° 33,18' N 11°17,83' E), unknown depth, 13.08.2013, coll. Arjan Gittenberger, Niels Schrieken (FD 057, 11.1 mm in length, live). NE Atlantic, North Sea, Dogger Bank (55° 09,20’ N 03° 19,04’ E), 30 m depth, 27.06.2012, coll. Arjan Gittenberger, Niels Schrieken (FD 059, 14.3 mm in length, live). NE Atlantic, North Sea, Eastern Scheldt, Netherlands (51° 37,75' N 03°54,82' E), 2–12 m depth, 29.06.2013, coll. F.M.F. Driessen, (FD 064, 9.2 mm in length, live). NE Atlantic, Porthkerris, United Kingdom (50° 03,57' N 05°03,55' W), 8 m depth, 24.09.2013, coll. F.M.F. Driessen R. Dekker, M. Spierenburg (FD 066, 5.9 mm in length, live). NE Atlantic, North Sea, Eastern Scheldt, Netherlands (51° 37,75' N 03°54,82' E), 2–12 m depth, 29.06.2013, coll. F.M.F. Driessen, (FD 071, 11.0 mm in length, live). NE Atlantic, North Sea, Eastern Scheldt, Netherlands (51° 37,75' N 03°54,82' E), 2–12 m depth, 29.06.2013, coll. F.M.F. Driessen, (FD 073, 9.6 mm in length, live).NE Atlantic, Porthkerris, United Kingdom (50° 03,57' N 05°03,55' W), 12 m depth, 25.09.2013, coll. F.M.F. Driessen R. Dekker, M. Spierenburg, (FD 077, 6.1 mm in length, live). NE Atlantic, Porthkerris, United Kingdom (50° 03,57' N 05°03,55' W), 8 m depth, 24.09.2013, coll. F.M.F. Driessen R. Dekker, M. Spierenburg, (ZMMU Op-758, 3.0 mm in length, live). NE Atlantic, Porthkerris, United Kingdom (50° 03,57' N 05°03,55' W), 8 m depth, 24.09.2013, coll. F.M.F. Driessen R. Dekker, M. Spierenburg, (FD 081, 8.9 mm in length, live). NE Atlantic, North Sea, Eastern Scheldt, Netherlands (51° 37,75' N 03°54,82' E), 2–12 m depth, 29.06.2013, coll. F.M.F. Driessen, (FD 083, 12.5 mm in length, live). NE Atlantic, North Sea, Eastern Scheldt, Netherlands (51° 37,75' N 03°54,82' E), 2–12 m depth, 29.06.2013, coll. F.M.F. Driessen, (FD 088, 12.4 mm in length, live). NE Atlantic, North Sea, Eastern Scheldt, Netherlands (51° 37,75' N 03°54,82' E), 2–12 m depth, 29.06.2013, coll. F.M.F. Driessen, (FD 094, 9.3 mm in length, live). NE Atlantic, North Sea, Eastern Scheldt, Netherlands (51° 37,75' N 03°54,82' E), 2–12 m depth, 29.06.2013, coll. F.M.F. Driessen, (FD 095, 16.0 mm in length, live). NE Atlantic, Lillebælt, Lyngsbo Strand, Denmark (55° 31,39' N 09°34,32' E), 2–4 m depth, 14.12.2013, coll. Ari J., (FD 101, 10 mm in length, live). NE Atlantic, Lillebælt, Lyngsbo Strand, Denmark (55° 31,39' N 09°34,32' E), 2–4 m depth, 14.12.2013, coll. J. Ari, (FD 102, 6.0 mm in length, live). NE Atlantic, Lillebælt, Lyngsbo Strand, Denmark (55° 31,39' N 09°34,32' E), 2–4 m depth, 14.12.2013, coll. J. Ari, (FD 103, 20 mm in length, live). NE Atlantic, Lillebælt, Lyngsbo Strand, Denmark (55° 31,39' N 09°34,32' E), 2–4 m depth, 14.12.2013, coll. J. Ari, (ZMMU Op-759, 12 mm in length, live). NE Atlantic, Lillebælt, Lyngsbo Strand, Denmark (55° 31,39’ N 09°34,32’ E), 2–4 m depth, 14.12.2013, coll. J. Ari, (FD 105, 9 mm in length, live). NE Atlantic, Lillebælt, Lyngsbo Strand, Denmark (55° 31,39’ N 09°34,32’ E), 2–4 m depth, 14.12.2013, coll. J. Ari, (FD 106, 15 mm in length, live). NE Atlantic, Vigo, Spain, 5 m depth, 03.09.2010, coll. T.A. Korshunova (ZMMU Op-368, 6 mm in length, preserved). NE Atlantic, Sagres, Portugal (37° 0,43' N 8°55,52' W), 16 m depth, 08.08.2019, B.E. Picton, (ZMMU Op-760, 12 mm in length, live). NE Atlantic, Sagres, Portugal 37° 0,43' N 8°55,52' W), 16 m depth, 08.08.2019, coll. B.E. Picton, (ZMMU Op-761, 12 mm in length, live). NE Atlantic,

Sagres, Portugal 37° 0,43' N 8°55,52' W), 16 m depth, 08.08.2019, coll. B.E. Picton, (ZMMU Op-762, 9 mm in length, live). NE Atlantic, Armação de Pêra, Portugal (37° 2,55' N 8°19,90' W), 18 m depth, 11.08.2019, coll. B.E. Picton, (ZMMU Op-763, 11 mm in length, live).

Diagnosis. Max. length 45 mm. Notal edge absent. Mean 11–12 rhinophoral lamellae (max. 17). Mean 4–5, max 7 frontal veil appendages. Mean 6–7, max. 11 gills. Postbranchial lobes simple, one pair (this structure has been termed in various sources as processes, papillae, or "ceras") rarely bifurcate. Chromatic variant I had not been found in adults, variants IV–VIII with variously expressed blackish stripes. Radular formula commonly 9–15 x 0–5.1.1.0.1.1.0–5 (max. 20 rows of teeth and five outer lateral teeth). First inner lateral teeth smaller and different in shape compared to larger second lateral teeth. Middle cusp of first lateral teeth distinct. Ampulla relatively large, bent in middle part. Bursa copulatrix large, elongate. Vas deferens considerably widens towards penial sheath. Two main types of copulative spines: shorter more elongate cones and very long, straight or winding spines, some of them bifurcated.

Description. *External morphology* (Figures 1, 2, 5, 10). Live length of neotype 25 mm. Length of adult specimens may reach 45 mm, usually 15–30 mm. Notum narrow, notal edge absent. Rhinophores without sheaths. 10–17 (usually 11–12 in present material) rhinophoral lamellae. Oral veil trapezoid, with oblique notched lateral sides. Anterior notum forms frontal veil with 4–7 elongated appendages (usually 4 in present material). Notum smooth or covered with low to slightly pointed tubercles. Spicules form sparse network in notum. Commonly 6–7 (max. 11) unipinnate or partially multipinnate gills united with a joint membrane into circle around anus. Gill cavity absent. One pair of simple (rarely bifurcate, possibly as a result of regeneration) postbranchial lobes. Foot narrow, anteriorly with pointed corners and thickened to form a double edge with a middle notch; posteriorly forming a long-pointed tail.

Chromatic variants (see Figure 1). Variant I has not been found. Variant II with orange-yellow spots. Variant III with distinct orange-yellow lines and spots. Variant IV with very weak black stripes in addition to orange-yellow spots. Variant V with few black stripes in addition to orange-yellow spots. Variant VI with evident black stripes in addition to orange-yellow spots. Variant VII with dominant black stripes in addition to orange-yellow spots. Variant VIII with dominant black stripes and distinct orange-yellow (which often became reddish) lines.

Internal morphology. Jaws with moderately narrow shoulders and strong, distinct wing-like expansions (Figure 5E, F). Radular formula (Figure 5C, I, N, T): 15 x 1–5.1.1.0.1.1.0–5 (neotype), 11 x 0–4.1.1.0.1.1.0–4 (Portugal, ZMMU Op-760), 9 x 0–4.1.1.0.1.1.0–4 (Portugal, Op-762), 9 x 1–5.1.1.0.1.1.1–5 (UK, Op-757), 9 x 0–4.1.1.0.1.1.0–4 (UK, ZMMU Op-756), 10 x 0–4.1.1.0.1.1.0–4 (UK, ZMMU Op-758). First inner lateral teeth smaller and different in shape compare to larger second lateral teeth. Middle cusp of first lateral teeth distinct. One to five outer lateral teeth, commonly maximum number four to five, the outer lateral teeth can be completely absent in the anterior most rows (Figure 5C, I).

Reproductive system (Figure 6A). Ampulla relatively large, conspicuously bent in middle part. Ampulla bifurcates into relatively long vas deferens and oviduct. Vagina narrow and enters large, elongate bursa copulatrix. Uterine duct relatively long and narrow; it begins from female gland mass and then enters near base of a medium-sized oval receptaculum seminis. Prostate large, wide, transiting to long, narrow vas deferens, which distinctly widens towards penial sheath that encloses eversible ejaculatory duct with copulative spines. Two main types of copulative spines detected: shorter more elongate cones and very long, straight needle-shaped or winding spines (Figure 5K, L, Q, R, V–X). Few more somewhat bent spines may present, but in *P. quadrilineata* never consistently hook-shaped throughout the ejaculatory duct, as in *P. capitata* (see below, Figure 7E, F, L, R, S, W–Z).

Ecology and distribution. *Polycera quadrilineata* is a common shallow-water species, usually recorded at depth *c.* 0–20 m, on stony substrates and kelps. Because previously several species were misidentified under the name "*P. quadrilineata* ", the verified food objects for true *P. quadrilineata* are bryozoans *Membranipora membranacea* (L., 1767) and *Scrupocellaria* spp. The verified geographic distribution includes Denmark, France, Germany, the Netherlands, Norway, Germany, Portugal, Spain, Sweden, and the Untied Kingdom (Figure 4). It was also recently recorded for the first time in the Arctic Barents Sea (Russia) due the climatic changes (Martynov *et al.* 2006).

Remarks. *Polycera quadrilineata* is one of the oldest species names for the European nudibranch molluscs. It is described in the year 1776 (Muller 1776). Since then, there were numerous attempts to separate more species similar to *P. quadrilineata*. However all of them without a detailed morphological analysis were considered as synonyms of *P. quadrilineata* (Thompson & Brown, 1984). Using molecular data, Driessen et al. (2014) showed two lineages among European "*P. quadrilineata*" for the first time. Recently (Sørensen et al. 2020) repeated the study of 2014, but without reference to it, with addition of a more detailed morphological study. However (Sørensen et al. 2020) incorrectly assessed the species identity and distributional patterns of the other hidden lineage because of an incomplete taxonomic analysis (see details in the remarks for *P. capitata* and in Discussion). Further, in the present study we revealed considerable errors in the apparent distinguishing characters between *P. quadrilineata* and *P. capitata*. Particularly, as four outer lateral teeth are clearly mentioned for *P. capitata* (as *P. norvegica*) and five outer lateral teeth for *P. quadrilineata* (Sørensen et al. 2020). However, in the present study we discovered, that *P. capitata* (= *P. norvegica*) may also possess up to five outer lateral teeth (Figure 7V). Therefore, when *P. quadrilineata* possesses five outer lateral teeth (likely due to the fact that *P. quadrilineata* is a larger species than *P. capitata*) more commonly than *P. capitata,* it is inaccurate to indicate this as a major distinguishing character between these species. Even a sixth outer lateral (rudimentary) tooth was detected in one specimen of *P. capitata* in the present study. Similarly, the shape of the jaws ("labial cuticle") was indicated as having much weaker wings in *P. capitata* than in *P. quadrilineata* (Sørensen et al. 2020), but this is not confirmed in the present study, since both species have quite strong wings of the jaws (compare Figure 5O, P and Figure 7D), though some details are different. The shape and size of the copulative spines thus is the most reliable internal morphological difference between *P. quadrilineata* and *P. capitata* (compare Figure 5K, L, Q, R, V–X and Figure 7E, F, L, R, S, W–Z). In the present study we additionally improved information on the patterns of copulative spines compared to the data in (Sørensen et al. 2020). The chromatic variants IV – VIII are readily distinguished between *P. quadrilineata* and *P. capitata* (Figs 1, 5, 7) and allows to unambiguously diagnose both these species. The chromatic variants II–III are more difficult to distinguish between *P. capitata* and *P. quadrilineata* but potentially possible using mapping of the chromatic polymorphism in the periodic-like framework established here (Fig. 1). *P. faeroensis* externally differs from *P. quadrilineata* by presence of commonly compound postbranchial lobes (if postbranchial lobe appears as a single pair, the smaller tubercles are usually present in addition) and absence of the chromatic variants III –VII (variant VIII may be rarely present in *P. faeroensis*, but differs from *P. quadrilineata* by absence of the blackish stripes) (Figure 1). Internally, *P. quadrilineata* considerably differs from *P. faeroensis* by the radular and jaws patterns and shape of the copulative spines (compare Figure 5K, L, Q, R, V–X and Figure 8O–Q). It was mentioned (Sørensen et al. 2020) that *P. capitata* differs from *P. quadrilineata* by presence of two types of the copulative spines. However, in the present study, we found that both *P. quadrilineata* and *P. capitata* have two (and more if use a fine-scale differentiation) types of the spines, but despite that shape and size of both types of the spines are easily distinguished between *P. capitata* and *P. quadrilineata* (Figures 5Q, V and 7W–Y). *P. quadrilineata* externally differs from *P. kernowensis* sp. nov. by presence of commonly more

than seven appendages of the frontal veil and absence of the chromatic variants II –VIII. Internally, *P. quadrilineata* considerably differs from *P. kernowensis* sp. nov. by the shape of the copulative spines (Figure 5K, L, Q, R, V–X and 9Q–S). The rechecked and improved distinguishing morphological characters of all four species in the European *Polycera* species complex are given in Table 1.

Polycera capitata **(Alder & Hancock, 1854)**

(Figures 1–4, 6B, 7, 10, S1; Tables 1, 2, S1, S2)

Synonymy:
Thecacera capitata Alder & Hancock, 1854: 103, 1855: iv, pl. 46 (supplementary), fig. 19; Thompson & Brown, 1984: 69, pl. 18c.
Polycera norvegica Sørensen et al., 2020: 13–17, figs 8–10, syn. nov.

Type locality: St. Ives, Cornwall, UK.

Material examined. Lectotype. NE Atlantic, St. Ives, Cornwall, UK (50° 14' N 5° 29' W), 36.5 m depth, 09.1853, coll. Mr. Barlee (preserved length 6.5 mm, radula slide 3124107 in Great North Museum: Hancock). Other materials. NE Atlantic, Porthkerris, United Kingdom (50° 03,57' N 05°03,55' W), 8 m depth, 24.09.2013, coll. F.M.F. Driessen R. Dekker, M. Spierenburg (FD 002, 6.2 mm in length, live). NE Atlantic, Porthkerris, United Kingdom (50° 03,57' N 05°03,55' W), 8 m depth, 24.09.2013, coll. F.M.F. Driessen R. Dekker, M. Spierenburg (FD 003, 5.4 mm in length, live). NE Atlantic, Porthkerris, United Kingdom (50° 03,57' N 05°03,55' W), 12 m depth, 25.09.2013, coll. F.M.F. Driessen R. Dekker, M. Spierenburg (ZMMU Op-764, 6 mm in length, live). NE Atlantic, Porthkerris, United Kingdom (50° 03,57' N 05°03,55' W), 8 m depth, 24.09.2013, coll. F.M.F. Driessen R. Dekker, M. Spierenburg (ZMMU Op-765, 6.5 mm in length, live). NE Atlantic, Porthkerris, United Kingdom (50° 03,57' N 05°03,55' W), 12 m depth, 25.09.2013, coll. F.M.F. Driessen R. Dekker, M. Spierenburg (FD 013, 3.2 mm in length, live). NE Atlantic, Porthkerris, United Kingdom (50° 03,57' N 05°03,55' W), 12 m depth, 25.09.2013, coll. F.M.F. Driessen R. Dekker, M. Spierenburg (FD 016, 6.5 mm in length, live). NE Atlantic, Porthkerris, United Kingdom (50° 03,57' N 05°03,55' W), 8 m depth, 24.09.2013, coll. F.M.F. Driessen R. Dekker, M. Spierenburg (FD 017, 5.2 mm in length, live). NE Atlantic, North Sea, Dogger Bank (55° 09,20’ N 03° 19,04’ E), 33 m depth, 30.06.2012, coll. Arjan Gittenberger, Niels Schrieken (FD 018, 3.8 mm in length, live). NE Atlantic, Porthkerris, United Kingdom (50° 03,57' N 05°03,55' W), 8 m depth, 24.09.2013, coll. F.M.F. Driessen R. Dekker, M. Spierenburg (FD 021, 7.2 mm in length). NE Atlantic, Porthkerris, United Kingdom (50° 03,57' N 05°03,55' W), 15 m depth, 26.09.2013, coll. F.M.F. Driessen R. Dekker, M. Spierenburg (FD 025, 5.8 mm in length, live). NE Atlantic, North Sea, Dogger Bank (55° 09,20’ N 03° 19,04’ E), 31 m depth, 25.06.2012, coll. Arjan Gittenberger, Niels Schrieken (FD 029, 5.4 mm in length, live). NE Atlantic, Porthkerris, United Kingdom (50° 03,57' N 05°03,55' W), 8 m depth, 24.09.2013, coll. F.M.F. Driessen R. Dekker, M. Spierenburg (FD 031, 6.9 mm in length, live). NE Atlantic, Porthkerris, United Kingdom (50° 03,57' N 05°03,55' W), 15 m depth, 26.09.2013, coll. F.M.F. Driessen R. Dekker, M. Spierenburg (ZMMU Op-766, 4.9 mm in length). NE Atlantic, North Sea, Dogger Bank (55° 09,20’ N 03° 19,04’ E), 31 m depth, coll. Arjan Gittenberger, Niels Schrieken (FD 034, 4.5 mm in length, live). NE Atlantic, Porthkerris, United Kingdom (50° 03,57' N 05°03,55' W), 8 m depth, 24.09.2013, coll. F.M.F. Driessen R. Dekker, M. Spierenburg (FD 037, 6.4 mm in length, live). NE Atlantic, Porthkerris, United Kingdom (50° 03,57' N 05°03,55' W), 8 m depth, 24.09.2013, coll. F.M.F. Driessen R. Dekker, M. Spierenburg (FD 039, 7.3 mm in length). NE Atlantic, Porthkerris, United Kingdom (50° 03,57' N 05°03,55' W), 8 m depth, 24.09.2013, coll. F.M.F. Driessen R. Dekker, M. Spierenburg (FD

042, 6.5 mm in length, live). NE Atlantic, Porthkerris, United Kingdom (50° 03,57' N 05°03,55' W), 8 m depth, 24.09.2013, coll. F.M.F. Driessen R. Dekker, M. Spierenburg (FD 043, 6.5 mm in length, live). NE Atlantic, Porthkerris, United Kingdom (50° 03,57' N 05°03,55' W), 8 m depth, 24.09.2013, coll. F.M.F. Driessen R. Dekker, M. Spierenburg (FD 050, 5.2 mm in length, live). NE Atlantic, Porthkerris, United Kingdom (50° 03,57' N 05°03,55' W), 12 m depth, 25.09.2013, coll. F.M.F. Driessen R. Dekker, M. Spierenburg (FD 052, 4.9 mm in length, live). NE Atlantic, Porthkerris, United Kingdom (50° 03,57' N 05°03,55' W), 12 m depth, 25.09.2013, coll. F.M.F. Driessen R. Dekker, M. Spierenburg (FD 053, 6.3 mm in length, live). NE Atlantic, Porthkerris, United Kingdom (50° 03,57' N 05°03,55' W), 8 m depth, 24.09.2013, coll. F.M.F. Driessen R. Dekker, M. Spierenburg (FD 056, 5.6 mm in length, live). NE Atlantic, North Sea, Dogger Bank (55° 09,20’ N 03° 19,04’ E), 33 m depth, 30.06.2012, coll. Arjan Gittenberger, Niels Schrieken (FD 060, 3.7 mm in length, live). NE Atlantic, Porthkerris, United Kingdom (50° 03,57' N 05°03,55' W), 12 m depth, 25.09.2013, coll. F.M.F. Driessen R. Dekker, M. Spierenburg (FD 061, 6.0 mm in length, live). NE Atlantic, Porthkerris, United Kingdom (50° 03,57' N 05°03,55' W), 8 m depth, 24.09.2013, coll. F.M.F. Driessen R. Dekker, M. Spierenburg (FD 062, 7.2 mm in length, live). NE Atlantic, Porthkerris, United Kingdom (50° 03,57' N 05°03,55' W), 8 m depth, 24.09.2013, coll. F.M.F. Driessen R. Dekker, M. Spierenburg (FD 065, 5.9 mm in length, live). NE Atlantic, Porthkerris, United Kingdom (50° 03,57' N 05°03,55' W), 8 m depth, 24.09.2013, coll. F.M.F. Driessen R. Dekker, M. Spierenburg, (FD 067, 5.7 mm in length, live). NE Atlantic, North Sea, Dogger Bank (55° 09,20’ N 03° 19,04’ E), 31 m depth, 25.06.2012, coll. Arjan Gittenberger, Niels Schrieken (FD 068, 6.3 mm in length, live). NE Atlantic, North Sea, Dogger Bank (55° 09,20’ N 03° 19,04’ E), 31 m depth, 25.06.2012, coll. Arjan Gittenberger, Niels Schrieken (FD 070, 6.6 mm in length, live). NE Atlantic, North Sea, Dogger Bank (55° 09,20’ N 03° 19,04’ E), coll. Arjan Gittenberger, Niels Schrieken (FD 072, 5.7 mm in length, live). NE Atlantic, Porthkerris, United Kingdom (50° 03,57' N 05°03,55' W), 8 m depth, 24.09.2013, coll. F.M.F. Driessen R. Dekker, M. Spierenburg (FD 074, 7.9 mm in length, live). NE Atlantic, Mullaghmore, Sligo, Ireland (54° 28,21' N 8°26,76' W), 5 m depth, 30.07.2019, coll. B.E. Picton (ZMMU Op-767, 16 mm in length, live). NE Atlantic, North Sea, Dogger Bank (55° 09,20’ N 03° 19,04’ E), coll. Arjan Gittenberger, Niels Schrieken (FD 075, 4.8 mm in length, live). NE Atlantic, Mullaghmore, Sligo, Ireland (54° 28,21' N 8°26,76' W), 5 m depth, 30.07.2019, coll. B.E. Picton (ZMMU Op-768, 14 mm in length, live). NE Atlantic, Mullaghmore, Sligo, Ireland (54° 28,21' N 8°26,76' W), 5 m depth, 30.07.2019, coll. B.E. Picton (ZMMU Op-769, 13 mm in length, live). NE Atlantic, Mullaghmore, Sligo, Ireland (54° 28,21' N 8°26,76' W), 5 m depth, 30.07.2019, coll. B.E. Picton (ZMMU Op-770, 10 mm in length, live). NE Atlantic, Mullaghmore, Sligo, Ireland (54° 28,21' N 8°26,76' W), 5 m depth, 30.07.2019, coll. B.E. Picton (ZMMU Op-771, 9 mm in length, live). NE Atlantic, Mullaghmore, Sligo, (54° 28,21' N 8°26,76' W), 5 m depth, 30.07.2019, coll. B.E. Picton (ZMMU Op-772, 12 mm in length, live). NE Atlantic, Mullaghmore, Sligo, Ireland (54° 28,21' N 8°26,76' W), 5 m depth, 30.07.2019, coll. B.E. Picton (ZMMU Op-773, 11 mm in length, live).

Diagnosis. Max. length 16 mm. Notal edge absent. Mean 8 rhinophoral lamellae (max. 11). Mean 4–5, max. 6 frontal veil appendages. Mean 6–7, max. 9 gills. Postbranchial lobes simple, one pair. Chromatic variant I and II are not known in adults, variants IV–VIII with variously expressed brownish to blackish small spots. Radular formula commonly 9–10 x 0–5.1.1.0.1.1.0–5 (max. 10 rows of teeth and five outer lateral teeth). First inner lateral teeth smaller and different in shape compare to larger second lateral teeth. Middle cusp of first lateral teeth distinct. Ampulla relatively small, enlarged proximally. Bursa copulatrix medium-sized, oval. Vas deferens does not widen towards penial sheath. Two main types of copulative spines: more elongate ones and smaller distinctly hook-shaped spines (those dominate).

Description. *External morphology* (Figures 1, 2, 7, 10). Preserved length of lectotype 6.5 mm. Length of adult specimens may reach 16 mm. Notum narrow, notal edge absent. Rhinophores

without sheaths. 7–11 (commonly 8–9 in present material) rhinophoral lamellae. Notum smooth or covered with low to slightly pointed tubercles. Spicules form sparse network in notum. Commonly 6–7 (max. 9) multipinnate or partially multipinnate gills united by common membrane into circle around anus. Gill cavity absent. One pair of simple postbranchial lobes. Oral veil trapezoid, with oblique notched lateral sides. Anterior notum forms frontal veil with 4–6 elongated appendages (commonly 4 in present material). Foot narrow, anteriorly with pointed corners and slightly thickened to form double edge; it appears as entire or slightly notched in the middle; posteriorly forming a long-pointed tail.

Chromatic variants (according to Figure 1). Variant I had not found in adults. Variant II with orange-yellow spots. Variant III with orange-yellow spots, which tend to be less numerous and less elongated, than in *P. quadrilineata*. Variant IV with very weak orange-yellow spots and small dispersed brownish/blackish spots. Variant V with evident brownish/blackish small spots mixed with small orange-yellow spots. Variant VI with faint stripe-like brownish/blackish pattern without or with weak small orange-yellow spots. Variant VII with dominant brownish/blackish colouration without or with weak small orange-yellow spots. Variant VIII had not found.

Internal morphology. Jaws with moderately narrow shoulders with distinct wing-like expansions, delicate (Figure 7D, K). Radular formula (Figure 7B, C, I, J, P, Q, V): 9 x 1–4.1.1.0.1.1.1–4 (Ireland, ZMMU Op-769), 9 x 0–5.1.1.0.1.1.0–5(6?) (Ireland, ZMMU Op-771), 10 x 3–4.1.1.0.1.1.3–4 (Ireland, ZMMU Op-773), 10 x 1– 5.1.1.0.1.1.1–5 (Ireland, ZMMU Op-767), 10 x 2– 4.1.1.0.1.1.2–4 (Ireland, ZMMU Op-768), 9 x 1– 5.1.1.0.1.1.1–5 (Ireland, ZMMU Op-770), 10 x 2– 4.1.1.0.1.1.2–4 (UK, ZMMU Op-764), 10 x 0–4.1.1.0.1.1.0–4 (UK, ZMMU Op-765), 10 x 1– 4.1.1.0.1.1.0–4 (UK, ZMMU Op-766). First inner lateral teeth smaller and different in shape compared to the larger second lateral teeth. Middle cusp of first lateral teeth distinct. One to five outer lateral teeth, commonly maximum number four, five appears as rarer, the outer lateral teeth can be completely absent in anterior most rows (Figure 7I, Q).

Reproductive system (Figure 6B). Ampulla relatively small, somewhat enlarged in the proximal part. Ampulla bifurcates into relatively long vas deferens and oviduct. Vagina narrow and enters medium-sized, widened oval bursa copulatrix. Uterine duct relatively long and narrow; it begins from female gland mass and then enters near base of a medium-sized oval receptaculum seminis. Prostate large, wide, transits to long, narrow vas deferens, which not considerably widens towards penial sheath that encloses eversible ejaculatory duct with copulative spines. Two types of copulative spines detected: more elongate ones, and shorter, distinctly hook-shaped spines (Figure 7E, F, L, R, S, W–Z). Few more somewhat bent spines may present in *P. quadrilineata* (Figure 5V), but in *P. capitata* spines consistently hook-shaped throughout almost all ejaculatory duct (Figure 7W–Y).

Ecology and distribution. *Polycera capitata* is a common shallow-water species, usually recorded at depth *c.* 2–40 m, on stony substrates and kelps. The verified food object is bryozoan *Electra pilosa* (L., 1767). The verified geographic distribution includes Ireland, the Netherlands, Norway, and the Untied Kingdom (Figure 4).

Remarks. Since the first description of "*P. quadrilineata*" several chromatic variants have been recognized, to which particular taxonomic names were assigned. A hidden lineage within *P. quadrilineata* was detected (Driessen et al. 2014), but not formally named. In 2020 for this lineage "*P. norvegica*" was proposed (Sørensen et al. 2020), however a valid synonym of *P. capitata* (Alder & Hancock, 1854) was overlooked and omitted (see details in Discussion). In the present study we therefore restored the species *P. capitata* and *P. norvegica* became its junior synonym. Here using the mapping of the chromatic variants and statistical analysis of the

diagnostic characters we revealed that *P. capitata* and *P. quadrilineata* are not only considerably different in the internal characters, but also reveal statistically significant differences in the number of rhinophoral lamellae in mature specimens: mean 8 in *P. capitata* and 11–12 in *P. quadrilineata*. Moreover, adult specimens of *P. capitata* are generally considerably smaller (known up to 16 mm length) than *P. quadrilineata* (up to 45 mm length). The majority of the chromatic variants of *P. capitata* (Figure 1, IV-VII) are quite distinct from the respective variants in *P. quadrilineata* (Figure 1). The specimens of chromatic variants II and III, which are similar between *P. capitata* and *P. quadrilineata,* can be reliably distinguished using the number of rhinophoral lamellae (see above in the *P. quadrilineata* remarks). The internal differences between *P. capitata* and *P. quadrilineata* are very solid ones (compare Figures 5, 7) and easily distinguish these species using the shape of the copulative spines (compare Figures 5K, L, Q, R, V–X and Figures 7E, F, L, R, S, W–Z). Notably, fine morphologists yet in the 19th century [Bergh, 1880] using light microscopy were able to clearly depict very long, winding copulative spines for true *P. quadrilineata* (Figures 5R) and hence if they would have had the opportunity to study *P. capitata* in detail, even without application of the molecular data, they would be able to distinguish the considerably different copulative spines of *P. capitata* (the majority of them appeared instead as small hooks), which in the present study are studied using scanning electron techniques (Figures 7E, F, L, R, S, W–Z). Therefore, the recent notion that *P. capitata* is a "cryptic species" (Sørensen et al. 2020) is incorrect (see Discussion). From *P. faeroensis* and *P. kernowensis* sp. nov., *P. capitata* differs in a number of features, such as the statistically significantly larger number of rhinophoral lamellae (mean 18–19 in *P. faeroensis*, 14–15 in *P. kernowensis* sp. nov., 8 in *P. capitata*) and frontal veil appendages (mean 9–10 in *P. faeroensis*, 7–8 in *P. kernowensis* sp. nov., 4 in *P. capitata*) even in smaller specimens (Figures 1, 2, S1), by absence of the chromatic variant I in *P. capitata* and by the internal characters (compare Figures 7–9). The rechecked and improved distinguishing morphological characters of all four species in the European *Polycera* species complex are given in Table 1.

Polycera faeroensis **Lemche, 1929**

(Figures 1–4, 6C, 8, S1; Tables 1, 2, S1, S2)

Synonymy:

Polycera faeroensis Lemche, 1929: 12–15, figs 1, 2; Odhner, 1941: 3–7, figs 1–4.

Non *Polycera faeroensis* sensu Lemche & Thompson, 1974, Thompson & Brown, 1984 (except for the radula description (p. 68) which mainly refers to the true *P. faeroensis*) and auctorum (mixture with *Polycera kernowensis* sp. nov., see below).

Type locality: the Faeroe Islands.

Holotype GAS-001512 in Natural History Museum of Denmark [NHMD, 2020].

Material examined. NE Atlantic, North Sea, Dogger Bank (55° 09,20' N 03° 19,04' E), 29 m depth, 26.06.2012, coll. Arjan Gittenberger, Niels Schrieken (FD 028, 21 mm in length, live). NE Atlantic, Belfast Lough, Northern Ireland (54° 41,00' N 5° 37,83' W), 20 m depth, coll. B.E. Picton, 19.07.2019 (ZMMU Op-774, 30 mm in length, live). Verified photographic records. NE Atlantic, Porthkerris, Cornwall, England (50° 20,04' N 5° 14,03' W), 10 m depth, B.E. Picton, 13.07.2013 (18 mm in length, live). NE Atlantic, Strangford Lough, Northern Ireland (54° 24,15' N 5° 36,57' W), 18 m depth, B.E. Picton, 20.05.2014 (22 mm in length, live). NE Atlantic, Strangford Lough, Northern Ireland (54° 23,37' N 5° 34,39' W), 10 m depth, B.E. Picton, 21.05.2014 (20 mm in length, live). NE Atlantic, Strangford Lough, Northern Ireland (54° 23,93' N 5° 36,82' W), 22 m depth, B.E. Picton, 23.05.2014 (25 mm in length, live). NE Atlantic,

Strangford Lough, Northern Ireland (54° 23,60' N 5° 36,33' W), 17 m depth, B.E. Picton,13.05.2015 (21 mm in length, live). NE Atlantic, Isle of Man (54° 3,77' N 4° 40,70' W), 18 m depth, B.E. Picton, 14.06. 2015 (22 mm in length, live). NE Atlantic, Isle of Man (54° 3,77' N 4° 40,70' W), 22 m depth, B.E. Picton, 14.06.2015 (24 mm in length, live, feeding on *Scrupocellaria scruposa*). NE Atlantic, Isle of Man (54° 3,62' N 4° 48,91' W), 25 m depth, coll. B.E. Picton, 16.06.2015 (20 mm in length, live). NE Atlantic, Skomer Island, Pembrokeshire, Wales (51° 43,99' N 5° 16,39' W), 14 m depth, B.E. Picton, 21.06.2018, mating pair (23 and 22 mm in length, live). NE Atlantic, Skomer Island, Pembrokeshire, Wales (51° 43,86' N 5° 18,07' W), 21 m depth, B.E. Picton, 22.06.2018 (25 mm in length, live). NE Atlantic, Rathlin Island, Northern Ireland (55° 17,82' N 6° 17,34' W), 24 m depth, B.E. Picton, 14.05.2019 (24 mm in length, live). NE Atlantic, Rathlin Island, Northern Ireland (55° 15,95' N 6°10,45' W), 31 m depth, B.E. Picton, 15.05.2019 (23 mm in length, live). NE Atlantic, Copeland Islands, Northern Ireland (54° 41,72' N 5° 30,67' W), 18 m depth, B.E. Picton, 04.07.2019, mating pair (23 and 25 mm in length, live). NE Atlantic, Bola Reef, Pembrokeshire, Wales (51° 58,32' N 5° 8,10' W), 25 m depth, coll. B.E. Picton, 05.08.2009 (30 mm in length, live). NE Atlantic, Rathlin Island, Northern Ireland (55° 17,54' N 6° 14,49' W), 28 m depth, B.E. Picton, date 07.06.2006 (25 mm in length, live, feeding on *Caberea ellisi*). NE Atlantic, Rathlin Island, Northern Ireland (55° 17,46' N 6° 15,18' W), 30 m depth, B.E. Picton, 09.06.2006 (30 mm in length, live). NE Atlantic, Rathlin Island, Northern Ireland (55° 17,46' N 6° 15,18' W), 30 m depth, B.E. Picton, 09.06.2006 (25 mm in length, live). NE Atlantic, Strangford Lough, Northern Ireland (54° 26,77' N 5° 35,26' W), 18 m depth, B.E. Picton, 23.05.2007 (23 mm in length, live). NE Atlantic, Strangford Lough, Northern Ireland (54° 24,90' N 5° 36,63' W), 14 m depth, B.E. Picton, 31.05.2007 (30 mm in length, live). NE Atlantic, Strangford Lough, Northern Ireland (54° 25,43' N 5° 34,87' W), 12 m depth, B.E. Picton, 05.06.2007 (22 mm in length, live). NE Atlantic, Rathlin Island, Northern Ireland (55° 18,28' N 6° 16,68' W), 30 m depth, B.E. Picton, 18.06.2007 (30 mm in length, live). NE Atlantic, Belfast Lough, Northern Ireland (54° 45,94' N 5° 40,69' W), 23 m depth, B.E. Picton, 13.05.1981 (25 mm in length, live). NE Atlantic, Sark, Channel Isles (49° 24,09' N 2° 21,51' W), 24 m depth, Jen Jones, 23.06.2010 (30 mm in length, live).

Diagnosis. Max. length 45 mm. Notal edge rudiments partially present anteriorly beyond the frontal veil and posteriorly around gills. Mean 18–19 rhinophoral lamellae (max. 25). Mean 9–10, max. 14 frontal veil appendages. Mean 6–7, max. 11 gills. Postbranchial lobes commonly with 2–5 main pairs (plus several smaller in addition; rarely a single larger lobe with a few small tubercles) and form a compound ridge (rudiments of notum). Chromatic variants I and II dominate in adults, variants III–VI and VIII had not found, rarely variant VIII occurs. Radular formula commonly 11–16 x 0–3.1.1.0.1.1.0–3 (max. 16 rows of teeth and three outer lateral teeth). First inner lateral teeth do not differ in shape and size compared to the larger second lateral teeth. Middle cusp of first lateral teeth not evident. Ampulla relatively large, bent. Bursa copulatrix very large, elongate. Vas deferens not widening towards penial sheath. Two main types of copulative spines: more elongate, strong cones (which dominate) and shorter with widened base, smaller hook-shaped or needle-shaped spines absent.

Description. *External morphology* (Figures 1, 2, 8). Preserved length of holotype 25 mm. Length of adult live specimens may reach 45 mm. Notum narrow, notal edge present, considerably reduced. Rhinophores without sheaths. 15–25 (commonly 18–20 in present material) rhinophoral lamellae. Notum smooth. Commonly 6–7, max 11 unipinnate or partially multipinnate gills united by a common membrane into a circle around anus. Gill cavity absent. Commonly 2–5 pairs of post branchial lobes (plus some smaller appendages; rarely a single larger lobe with few small tubercles) form a conspicuous compound ridge which represents remnants of the notal edge. Oral veil trapezoid, with oblique notched lateral sides. Anterior notum forms frontal veil with 9 to 14 elongated appendages of different length. Foot narrow,

anteriorly with pointed corners and slightly thickened to form a double edge; it appears as entire or slightly notched in the middle; posteriorly it forms a long-pointed tail.

Chromatic variants (see Figure. 1). Variant I commonly present in adults. Variants II and III more rarely occur, orange-yellow spots restricted mostly to the lateral sides, if present. Variants IV–VII are not known. Variant VIII with dark dominant colouration (but without any black stripes) was encountered for two specimens (not sequenced). The variant morphologically fits well to the diagnosis of *P. faeroensis* (by presence of compound postbranchial lobes with up to three separate processes).

Internal morphology. Jaws with broad, rounded shoulders, without distinct wing-like expansions (Figure 8M, N). Radular formula (Figure 8I, J–L): 11 x 0–3.1.1.0.1.1.0–3 (Ireland, ZMMU Op-774), 16 x 2.1.1.0.1.1.2 (holotype, the Faeroe Islands, Lemche, 1929), 16 x 3.1.1.0.1.1.3 (Sweden, Odhner, 1941). First inner lateral teeth similar in size to the second lateral teeth. Middle cusp of first lateral teeth not evident. Outer lateral teeth max 3.

Reproductive system (Figure 6C). Ampulla relatively large, conspicuously bent in middle part. Ampulla bifurcates into very long vas deferens and oviduct. Vagina narrow and enters very large, elongate bursa copulatrix. Uterine duct relatively long and narrow; it begins from female gland mass and then enters near base of a very large oval elongated receptaculum seminis. Prostate large, wide, transits to a very long, narrow vas deferens, which not widens towards penial sheath that encloses the avertable ejaculatory duct with copulative spines. Copulative spines (Figure 8O–Q). At least two types detected: shorter more elongate cones with a peculiar base with a hole and long, straight or slightly bent spines, which differs in details from all other three species, *P. quadrilineata*, *P. capitata* and *P. kernowensis* sp. nov. (compare Figure 5K, L, Q, R, V–X, Figure 7E, F, L, R, S, W–Z, Figure 8O–Q and Figure 9Q–S).

Ecology and distribution. *Polycera faeroensis* is a relatively rare species in shallow waters, more commonly found in deeper localities at depth *c.* 20–100 m, on stony and muddy substrates. The verified food objects are bryozoans *Caberea ellisi* (Fleming, 1814) and *Scrupocellaria* spp. The verified geographic distribution includes the Faeroe Islands, the Netherlands, Norway, Sweden and the Untied Kingdom (Figure 4; Lemche 1929; Odhner 1941).

Remarks. *Polycera faeroensis* is the one of the less studied and poorly understood species of the European *Polycera* complex because previously it was confused not only with its sister species *P. kernowensis* sp. nov. (Figures 1, 2, 8, 9), but also with *P. quadrilineata* (Thompson & Brown 1984; Just & Edmunds 1985; Sørensen et al. 2020). In the present study we verified its identification using a molecularly proven specimen that was also thoroughly studied morphologically for the first time (Figures 1, 3, 9). The shape and size of the lateral teeth of the radula in the studied molecularly proven specimen (Figures 9J –L) exactly matched with the pattern described for the holotype of *P. faeroensis* and additional specimens from Sweden as described in (Lemche, 1929 and Odhner 1941). However the radulae of specimens mistakenly identified as "*P. faeroensis*" in (Lemche & Thompson 1974; Thompson & Brown 1984, Figure 9C) are considerably different both by its formula and shape of the teeth. The latter species was revealed in the present study as *P. kernowensis* sp. nov. (see below and Figures 1–3, 9). The number of the outer lateral teeth completely equals the number of outer lateral teeth in the holotype from the Faeroe Islands (Lemche 1929) and redescribed specimens from Sweden (Odhner, 1941). Our specimen and type specimen of *P. faeroensis* also share absence of evident middle cusp of first lateral teeth (compare Figure 8I, K) and this feature clearly distinguishes the radula of *P. faeroensis* from three other species within the European *Polycera* species complex. In addition to our own specimens of *P. faeroensis*, four specimens are present in the BOLD sequences as private data, which made it impossible to include to the present molecular

phylogenetic tree (Figures 1, 3). However, it is possible to compare these sequences using the BOLD identification tool, and all four specimens (SSSN527-19, SSSN361-19, SSSN352-19, and SSSN528-19) are well matched to our verified *P. faeroensis.* Further, available at BOLD photographs of these four specimens *P. faeroensis* (## 126014, 126027, 127727, and 127728) are closely matched to our data. Thus, currently at least six specimens of the rarer species *P. faeroensis* can be verified with both morphological and molecular data from distant geographic locations (UK, Ireland and Norway). Therefore, the type locality of *P. faeroensis* at the Faeroe Islands is in-between the locations from where we have molecularly proven specimens. The identity of true *P. faeroensis* is now confirmed for the first time, as the morphological data for our molecularly proven specimen of *P. faeroensis* very well matches the original description of *P. faeroensis* (Lemche, 1929) and its redescription (Odhner, 1941). In a recent study, *P. faeroensis* was incorrectly identified with sequences from another species (Sørensen et al. 2020), which in the present study is described as *P. kernowensis* sp. nov. (Figures 1–4, 9). *Polycera quadrilineata* "var. *nonlineata*" (Thompson, 1840) was referred by Thompson and Brown (1984) with a question mark as a synonym of species *P. faeroensis*. However, according to the first description of *P. quadrilineata* "var. *nonlineata*" (Thompson 1840) has pair of simple postbranchial lobes and orange-yellow spots on the body, and by this combination it is impossible to refer that variety neither to *P. faeroensis* nor to *P. kernowensis* sp. nov. We consider *P. quadrilineata* "var. *nonlineata*" as a synonym of *P. quadrilineata.*
In addition to the molecularly verified specimens, we observed several more specimens of *P. faeroensis* in similar locations in Ireland and UK, studying well-documented photographs of individuals that were not collected (Figure 8D–G). Since external morphological data of these specimens are essentially similar to the molecularly verified specimens of *P. faeroensis* (Figures 3, 8A,B), these additional data were used in the present study for the statistical evaluation of the diagnostic reliability of the number of the frontal veil appendages, rhinophoral lamellae and gills (Figures 2, S1) and therefore listed above in the studied materials section. On some photographs we observed a few specimens with thickened postbranchial lobes, which appeared as rather simple with only lower tubercles in addition (Figure 8C). There is need to further test the generality of reduced instead of complex compound postbranchial lobes in adult specimens of *P. faeroensis*. The rechecked and improved distinguishing morphological characters of all four species in the European *Polycera* species complex are given in Table 1.

***Polycera kernowensis* Driessen, Martynov, Picton, Dekker & Korshunova, sp. nov.**
(Figures 1–4, 6D, 9, S1; Tables 1, 2, S1, S2)

Polycera faeroensis sensu Lemche & Thompson, 1974 (partim., figs 2, 3a, text description mixture with true *P. faeroensis*); Thompson & Brown, 1984 (partim., figs 14a, 18e?; text description mixture with true *P. faeroensis*); Sørensen et al., 2020; non Lemche, 1929.

ZooBank registration: urn:lsid:-zoobank.org:act: 5C821EFD-FB12-49D5-A9C3-026A325F6D21

Type locality: Porthkerris, United Kingdom.

Material examined. Holotype. NE Atlantic, Porthkerris, United Kingdom (50° 03,39' N 05°03,55' W), 12 m depth, 25.09.2013, coll. F.M.F. Driessen R. Dekker, M. Spierenburg (ZMMU Op-755, 5.6 mm in length, live). Paratypes. NE Atlantic, Porthkerris, United Kingdom (50° 03,39' N 05°03,55' W), 8 m depth, 25.09.2013, coll. F.M.F. Driessen R. Dekker, M. Spierenburg (FD 001, 4.4 mm in length, live). NE Atlantic, Setubal, Portugal (38° 28' N 08°56'

W), ca. 12 m depth, 31.10.2013, M. Spierenburg (ZMMU Op-775, 3.9 mm in length, live). NE Atlantic, Porthkerris, United Kingdom (50° 03,39’ N 05°03,55’ W), 15 m depth, 26.09.2013, coll. F.M.F. Driessen R. Dekker, M. Spierenburg (FD 006, 4.5 mm in length, live, feeding on *Scrupocellaria scruposa*). NE Atlantic, Porthkerris, United Kingdom (50° 03,39’ N 05°03,55’ W), 15 m depth, 26.09.2013, coll. F.M.F. Driessen R. Dekker, M. Spierenburg (FD 009, 6.7 mm in length, live). NE Atlantic, Porthkerris, United Kingdom (50° 03,39’ N 05°03,55’ W), 15 m depth, 26.09.2013, coll. F.M.F. Driessen R. Dekker, M. Spierenburg (FD 010, 5.6 mm in length, live). NE Atlantic, Porthkerris, United Kingdom (50° 03,39’ N 05°03,55’ W), 15 m depth, 26.09.2013, coll. F.M.F. Driessen R. Dekker, M. Spierenburg (FD 011, 5.0 mm in length, live, feeding on *Scrupocellaria scruposa*). NE Atlantic, North Sea, Dogger Bank (55° 09,20’ N 03° 19,04’ E), 30 m depth, 27.06.2012, coll. Arjan Gittenberger, Niels Schrieken (FD 012, 13.9 mm in length, live). NE Atlantic, North Sea, Dogger Bank (55° 09,20’ N 03° 19,04’ E), 30 m depth, 28.06.2012, coll. Arjan Gittenberger, Niels Schrieken (FD 020, 13.6 mm in length, live). NE Atlantic, Porthkerris, United Kingdom (50° 03,39’ N 05°03,55’ W), 15 m depth, 26.09.2013, coll. F.M.F. Driessen R. Dekker, M. Spierenburg (FD 022, 6.1 mm in length). NE Atlantic, Porthkerris, United Kingdom (50° 03,39’ N 05°03,55’ W), 15 m depth, 26.09.2013, coll. F.M.F. Driessen R. Dekker, M. Spierenburg (FD 023, 8.5 mm in length, live, feeding on *Scrupocellaria scruposa*). NE Atlantic, Porthkerris, United Kingdom (50° 03,39’ N 05°03,55’ W), 15 m depth, 26.09.2013, coll. F.M.F. Driessen R. Dekker, M. Spierenburg (FD 024, 6.8 mm in length, live). NE Atlantic, Porthkerris, United Kingdom (50° 03,39’ N 05°03,55’ W), 15 m depth, 26.09.2013, coll. F.M.F. Driessen R. Dekker, M. Spierenburg (FD 026, 3.5 mm in length, live). NE Atlantic, Porthkerris, United Kingdom (50° 03,39’ N 05°03,55’ W), 15 m depth, 26.09.2013, coll. F.M.F. Driessen R. Dekker, M. Spierenburg (FD 032, 3.5 mm in length, live). NE Atlantic, Porthkerris, United Kingdom (50° 03,39’ N 05°03,55’ W), 15 m depth, 26.09.2013, F.M.F. Driessen R. Dekker, M. Spierenburg (FD 035, 6.1 mm in length, live, feeding on *Scrupocellaria scruposa*). NE Atlantic, Porthkerris, United Kingdom (50° 03,39’ N 05°03,55’ W), 12 m depth, 25.09.2013, coll. F.M.F. Driessen R. Dekker, M. Spierenburg (FD 036, 6.5 mm in length, live). NE Atlantic, Porthkerris, United Kingdom (50° 03,39’ N 05°03,55’ W), ca. 15 m depth, 26.09.2013, coll. F.M.F. Driessen R. Dekker, M. Spierenburg (FD 046, 7.1 mm in length, live). NE Atlantic, Porthkerris, United Kingdom (50° 03,39’ N 05°03,55’ W), ca. 15 m depth, 26.09.2013, coll. F.M.F. Driessen R. Dekker, M. Spierenburg (ZMMU Op-776, 1.2 mm in length, live). NE Atlantic, Porthkerris, United Kingdom (50° 03,39’ N 05°03,55’ W), 15 m depth, 6.09.2013, coll. F.M.F. Driessen R. Dekker, M. Spierenburg (FD 049, 5.5 mm in length, live). NE Atlantic, Porthkerris, United Kingdom (50° 03,39’ N 05°03,55’ W), 12 m depth, 25.09.2013, coll. F.M.F. Driessen R. Dekker, M. Spierenburg (FD 058, 6.2 mm in length, live). NE Atlantic, Porthkerris, United Kingdom (50° 03,57' N 05°03,55' W), 15 m depth, 26.09.2013, coll. F.M.F. Driessen R. Dekker, M. Spierenburg (FD 063, 7.5 mm in length, live). NE Atlantic, Porthkerris, United Kingdom (50° 03,39’ N 05°03,55’ W), 15 m depth, 26.09.2013, coll. F.M.F. Driessen R. Dekker, M. Spierenburg (FD 069, 7.3 mm in length, live). NE Atlantic, Porthkerris, United Kingdom (50° 03,57' N 05°03,55' W), 12 m depth, 25.09.2013, coll. F.M.F. Driessen R. Dekker, M. Spierenburg, (FD 076). NE Atlantic, Porthkerris, United Kingdom (50° 03,39’ N 05°03,55’ W), 12 m depth, 25.09.2013, F.M.F. Driessen R. Dekker, M. Spierenburg (FD 079, 3.7 mm in length, live). NE Atlantic, Porthkerris, United Kingdom (50° 03,39’ N 05°03,55’ W), ca. 15 m depth, 26.09.2013, coll. F.M.F. Driessen R. Dekker, M. Spierenburg (FD 085, 4.1 mm in length, live). NE Atlantic, Porthkerris, United Kingdom (50° 03,39’ N 05°03,55’ W), 15 m depth, 26.09.2013, coll. F.M.F. Driessen R. Dekker, M. Spierenburg (FD 086, 3.9 mm in length, live). NE Atlantic, Porthkerris, United Kingdom (50° 03,39’ N 05°03,55’ W), 15 m depth, 26.09.2013, coll. F.M.F. Driessen R. Dekker, M. Spierenburg (FD 087, 5.5 mm in length, live). NE Atlantic, Porthkerris, United Kingdom (50° 03,39’ N 05°03,55’ W), 15 m depth, 26.09.2013, coll. F.M.F. Driessen R. Dekker, M. Spierenburg (FD 089, 5.5 mm in length, live). NE Atlantic, Porthkerris, United Kingdom (50° 03,39’ N 05°03,55’ W), 15 m depth, 26.09.2013, coll. F.M.F. Driessen R. Dekker, M. Spierenburg (FD 091, 5.8 mm in length, live). NE Atlantic, Porthkerris, United Kingdom

(50° 03,39’ N 05°03,55’ W), 15 m depth, 26.09.2013, F.M.F. Driessen R. Dekker, M. Spierenburg (FD 093, 5.4 mm in length, live). NE Atlantic, Porthkerris, United Kingdom (50° 03,39’ N 05°03,55’ W), 15 m depth, 26.09.2013, coll. F.M.F. Driessen R. Dekker, M. Spierenburg (FD 100, 6.7 mm in length, live). Verified photographic records. NE Atlantic, Isle of Man (54° 3,77’ N 4° 40,70’ W), 24 m depth, coll. B.E. Picton, 14.06.2015 (20 mm in length, live). NE Atlantic, Killary Harbour, Ireland (53° 37,79’ N 9° 53,47’ W), 15 m depth, coll. B.E. Picton, 05.05.2017 (20 mm in length, live).

Etymology. The species epithet **“***kernowensis*” is derived from an old name for Cornwall, where *P. kernowensis* sp. nov. is commonly occurred.

Diagnosis. Max. length 20 mm. Notal edge absent. Mean 14–15 rhinophoral lamellae (max. 22). Mean 7–8, max. 9 frontal veil appendages. Mean 4–5, max. 7 gills. Postbranchial lobes simple, without additional smaller tubercles, one pair. Chromatic variants I and II are not known in adults, variants IV–VIII with variously expressed blackish stripes. Radular formula commonly 7–10 x 0–4.1.1.0.1.1.0–4 (max. 10 rows of teeth and four outer lateral teeth known presently). First inner lateral teeth smaller and different in shape compared to the larger second lateral teeth. Middle cusp of first lateral teeth partially evident only in posterior part of radula. Ampulla relatively small, not bent in middle part. Bursa copulatrix large, elongate. Vas deferens not widens towards penial sheath. Presently known one type of copulative spines: elongate, somewhat bent cones, shorter cones can be potentially present.

Description. *External morphology* (Figures 1, 2, 9). Live length of holotype 5.6 mm. Length of adult specimens may reach 20 mm. Notum narrow, notal edge absent. Rhinophores without sheaths. 12–22 (commonly 14–15 in the present material) rhinophoral lamellae. Notum smooth. Spicules form sparse network in notum. Commonly 4–5, max. 7 unipinnate or partially multipinnate gills united by common membrane into circle around anus. Gill cavity absent. One pair of simple postbranchial lobes. Oral veil trapezoid, with oblique notched lateral sides. Anterior notum forms frontal veil with 7–9 elongated appendages. Foot narrow, anteriorly with pointed corners and slightly thickened to form a double edge; it appears as entire or slightly notched in the middle; posteriorly it forms a long-pointed tail.

Chromatic variants (see Figure 1). Variant I had only found. Variants II–VIII are not known. Potentially variants II and VII can be found.

Internal morphology. Jaws with moderately narrow shoulders, with distinct wing-like expansions (Figure 9I, O, P). Radular formula (Figure 9F, G, H, L, M, N): 8 x 1–4.1.1.0.1.1.1–4 (holotype UK, ZMMU Op-755), 10 x 0–4.1.1.0.1.1.0–4 (Portugal, ZMMU Op-775), 11 x 4.1.1.0.1.1.4 (Thompson & Brown, 1984 incorrectly identified specimen (as *P. faeroensis*) of *P. kernowensis* sp. nov.). First inner lateral teeth smaller and different in shape compared to larger second lateral teeth. Middle cusp of first lateral teeth partially evident only in posterior part of radula (this character needs to be further tested in this species). One to four outer lateral teeth (Figure 9F, G, H, L, M, N).

Reproductive system (Figure 9D). Ampulla relatively small, not evidently bent in middle part. Ampulla bifurcates into relatively long vas deferens and oviduct. Vagina narrow and enters large, elongate bursa copulatrix. Uterine duct relatively long and narrow; it begins from female gland mass and then enters near base of a medium-sized oval receptaculum seminis. Prostate large, wide, transits to long, narrow vas deferens, which not widens considerably towards penial sheath that encloses avertable ejaculatory duct with copulative spines. One type of copulative spines surely detected (more diversity can be further discovered): elongate, somewhat hooked cones

(Figure 9Q–S), which differs in detail from *P. quadrilineata*, *P. capitata* and *P. faeroensis* (compare Figure 5K, L, Q, R, V–X, Figure 7E, F, L, R, S, W–Z, Figure 8O–Q and Figure 9Q–S).

Ecology and distribution. *Polycera kernowensis* sp. nov. is a common shallow-water species, usually recorded at depth *c.* 8–30 m, on stony and muddy substrates. The verified food objects are bryozoans *Bicellariella ciliata* (L., 1758) and *Scrupocellaria* spp. The verified geographic distribution includes the Netherlands, Portugal, and the Untied Kingdom (Figure 4).

Remarks. Throughout the history of nudibranch studies in Europe, *P. kernowensis* sp. nov. has been confused with *P. faeroensis* and was never taxonomically recognized (Lemche & Thompson 1974; Thompson & Brown 1984; Sørensen et al. 2020), despite the presence of significant external and internal differences (Figures 1, 2, 9; Table 1).
P. kernowensis sp. nov. considerably differs from *P. faeroensis* by the presence of simple and not compound postbranchial lobes (in some specimens of *P. faeroensis* a single massive lobe present, but still it had few additional tubercles, whereas *P. kernowensis* sp. nov. has a more slender lobe and always without additional tubercles). Moreover, *P. kernowensis* sp. nov. usually has less than nine appendages of the frontal veil (*P. faeroensis* has commonly more than nine frontal appendages), chromatic variants II-VIII are absent, differentiated smaller first lateral teeth are present, there are up to four (instead of up to three) outer lateral teeth, it has a shorter vas deferens and the species have a different shape of the ampulla and bursa copulatrix, and of the copulative spines (Figures 1, 2, 8, 9; Table 1). A specimen of "*P. faeroensis*" was figured in (Thompson & Brown, 1984) (reproduced here with permission, Figure 9C) and well matches externally to the here described *P. kernowensis* sp. nov. except for the presence of an additional tubercle on the postbranchial lobes. In a few specimens of true *P. faeroensis* with less expressed compound postbranchial lobes, usually more than one tubercle is present on a lobe in addition to the tip (Figure 8C) (as figured for a specimen of apparent *P. faeroensis* in Just and Edmunds, 1985). From *P. quadrilineata* and *P. capitata*, *P. kernowensis* sp. nov. differs significantly by more lamellae on the rhinophores (mean 14–15 in the newly detected species vs. 11–12 in *P. quadrilineata* and only 8 in *P. capitata*), by significantly more appendages on the frontal veil (mean 7–8 in *P. kernowensis* sp. nov. vs. 4 in *P. quadrilineata* and *P. capitata*) in absence of the chromatic variants II–VIII, shape of the ampulla and copulative spines (compare Figures 1, 2, 5, 7, 9). P. *kernowensis* sp. nov. additionally differs from *P. quadrilineata* by shape of the ampulla and absence of widened distal part of vas deferens, and from *P. capitata* by the large elongate bursa copulatrix (compare Figures 6A, B, D). From the other three species in the European *Polycera* complex, as well as any other known polycerids it significantly differs by the molecular phylogenetic data (Figures 3, 4). The rechecked and improved distinguishing morphological characters of all four species in the European *Polycera* species complex are given in Table 1.
Regarding the COI marker, the maximal intragroup distance within *P. kernowensis* sp. nov. is 2.33%. Minimal intergroup distances between *P. kernowensis* sp. nov. and *P. quadrilineata, P. capitata, P. faeroensis,* and *P.* sp. A are 10.79%, 8.66%, 5.47%, and 8.71% respectively (Table 2).

References

Alder, J. & Hancock, A. (1854). Notice of some new species of British Nudibranchiata. *Annual Magazine Natural History*, *14*, 102–105.

Alder, J. & Hancock A. (1855). *A monograph of the British nudibranchiate Mollusca: with figures of all the species*. Pt. 7, pp. 1-54, fam. 1, pls. 21a, 27; fam. 2, pls. 1, 2; fam. 3, pls. 38a, 45–48. Appendix, pp. i–xl. The Ray Society, London.

Anderson, J. & Picton, B. (2017). Scottish Nudibranchs. (Kindle Edition).

Bergh R. (1879). Beiträge zu einer Monographie der Polyceraden. I. *Verhandlungen kaiserliche –königliche zoologische –botanische Gesellschaft in Wien*, *29,* 599–652.

Dautzenberg, P. & Durouchoux, P. (1913). Les mollusques de la Baie de Saint-Malo. *Feu. des Jeun. Natural.*, 43, 1–24.

Fenwick, D. (2020). An educational resource to the diversity of marine life of Great Britain and Ireland, accessed through https://www.aphotomarine.com/.

Ihering H. v. (1886). Beiträge zur Kenntniss der Nudibranchien des Mittelmeeres. II. 4. Die Polyceraden. *Malakozoologische Blãtter*, *8*, 12–48.

Just, H., & Edmunds, M. (1985). North Atlantic nudibranchs (Mollusca) seen by Henning Lemche, with additional species from the Mediterranean and the north east Pacific. *Ophelia* suppl., *2*, 1–170.

Labbé, A. L. (1931). Les polyceradés de la Station du Croisic et description sommaire d'une espèce nouvelle: *Polycera salamandra*, nov. sp. *Bulletin de la Societe Zoologique de France*, *56*, 19–24.

Lemche, H. (1929). Gastropoda Opisthobranchiata. In: R. Sparck & S. L. Tuxen (Eds.). *Zoology of Faroes*, *3*, 1–35.

Martynov A.V., Korshunova T.A., Savinkin O.V. (2006). Shallow-water opisthobranch molluscs of the Murman coast of the Barents Sea, with new distributional data and remarks on biology. *Ruthenica*, 16, 59–72.

Montagu, G. (1804). Description of several marine animals found on the south coast of Devonshire. *Transactions of the Linnean Society of London 7*, 61–85.

Müller, O. F. (1776). *Zoologiae Danicae. Prodromus seu animalium Daniae et Norvegiae.* Hallageriis, Copenhagen.

Müller, O. F. (1779). *Zoologiae Danicae sev animalium Daniae et Norvegiae.* (Sumtibus Weygandinis, Copenhagen and Leipzig.

NHMD, 2020. *Polycera faeroensis* type GAS-001512. Accessed through http://www.daim.snm.ku.dk/digitized-type-collection-details?cat,_fa1b4c8b-2fe4-4c23-bc37-caa21b5c849e

Odhner, N. (1941). New polycerid nudibranchiate Mollusca and remarks on this family. *Göteborgs Kungl. Vetenskaps- och Vitterhets-Samhälles Handlingar*, *91*, 1–20.

Rathke, J. In: O. F. Müller. (1806). *Zoologie Danica, sev Animalium Daniae et Norvegiae rariorum ac minus notorum descriptiones et historia*, *ed. 3*. (N. Christensen, Copenhagen).

Risso, A. (1826). *Histoire naturelle des principales productions de l'Europe Méridionale et particulièrement de celles des environs de Nice et des Alpes Maritimes, 4*.

Thompson, T.E. & Brown, G.H. (1984). *Biology of opisthobranch molluscs. Vol. 2.* The Ray Society Publications.

Sars, M. (1839). Undersøgelser over nogle lavere dyrs udvikling. *Nyt Mag. Naturvidensk.* 2, 139–166.

Thompson W. (1840). Contributions towards a knowledge of the Mollusca Nudibranchia and Mollusca Tunicata of Ireland, with descriptions of some apparently new species of Invertebrata. *Annals of Magazine Natural History*, *5*, 84–102.

Thompson, T.E. (1958). The natural history, embryology, larval biology and post-larval development of *Adalaria proxima* (Alder and Hancock) (Gastropoda Opisthobranchia). *Philosophical Transactions of the Royal Society of London*, Series B, *242*, 1–58.

Statistical differences in external morphology in *Polycera* species of all identified color patterns

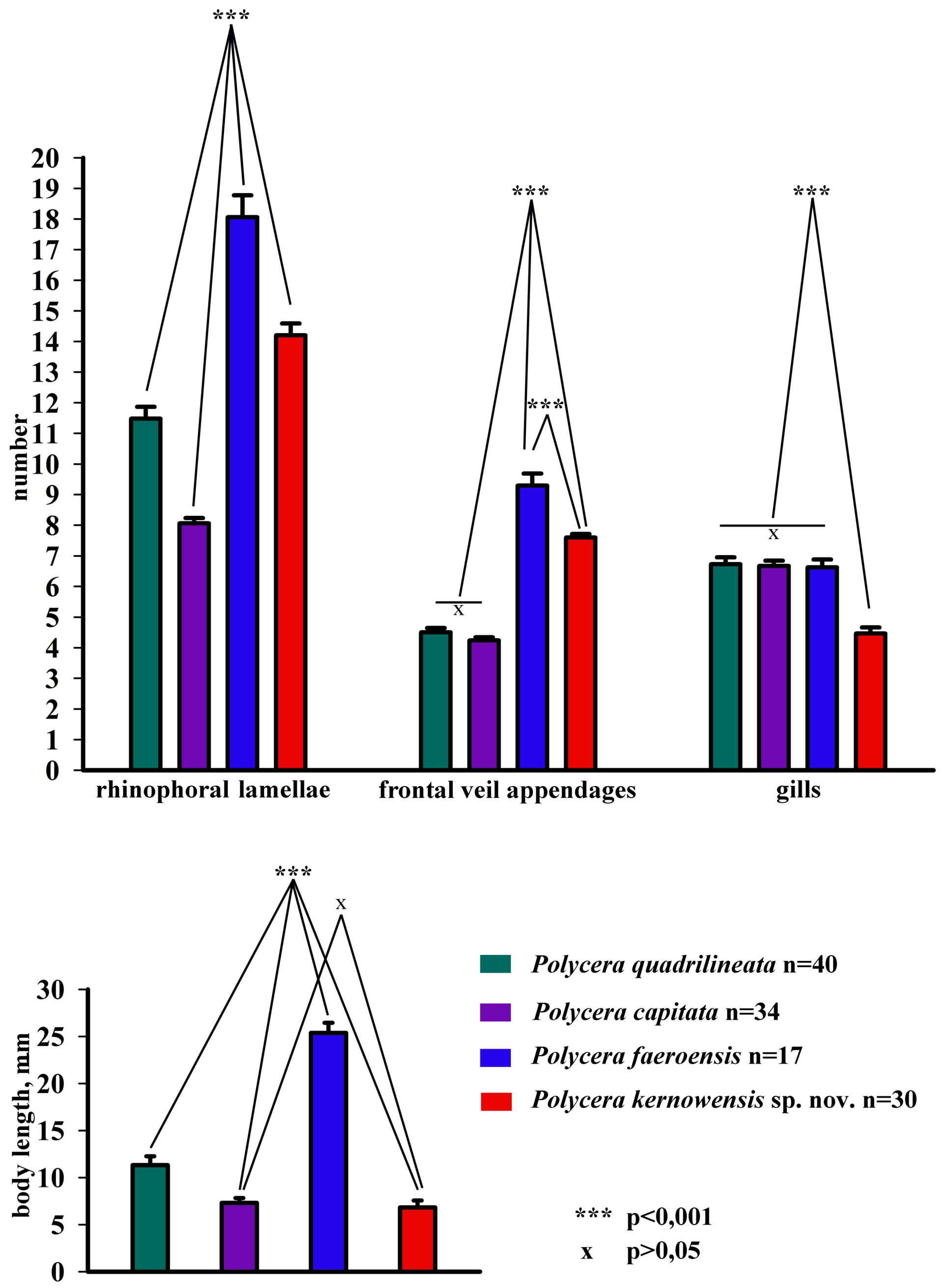

Supplementary information Figure S1. Statistical analysis of the external diagnostic characters among all identified colour patterns of European *Polycera* species (mean ± the standard error of the mean values).

Supplementary information Table S1. GenBank accession numbers for all sequences used in this study.

Species name	Voucher	Locality	COI	16S
Actinocyclus verrucosus Ehrenberg, 1831	CASIZ:189448	Hawaii	MF958438	MF958311
Hallaxa translucens Gosliner & S. Johnson, 1994	CAS:CASIZ:173447	Madagascar	EU982760	EU982814
Kalinga ornata Alder & Hancock, 1864	ZMMU Op-83	Vietnam	MN224072	MN224103
Kaloplocamus ramosus (Cantraine, 1835)	MNCN:15.05/55473	Australia: New South Wales	JX274104	JX274066
Kaloplocamus sp.	CASIZ:194412	Madagascar	MF958429	MF958299
Limacia clavigera (O. F. Müller, 1776)	-	Spain	EF142906	EF142952
Limacia sp.	CASIZ:176312	South Africa	HM162692	HM162602
Nembrotha cristata Bergh, 1877	CASIZ1:91428	Papua New Guinea	MF958431	MF958301
Nembrotha kubaryana Bergh, 1877	-	Taiwan?	NC_034920	NC_034920
Palio dubia (Sars, 1829)	ZMMU Op-751	Russia: White Sea	**MZ425304**	**MZ420394**
Palio dubia (Sars, 1829)	-	Sweden: Kristineberg, Bohuslän	AJ223272	AJ225197
Palio dubia (Sars, 1829)	MNCN:15.05/55467	Sweden: Gullmaren, Bohuslän	JX274100	-
Polycera alabe Collier & Farmer, 1964	CPIC00809	Mexico	-	KF425269
Polycera alabe Collier & Farmer, 1964	LACM:140739	Mexico	-	KF425268
Polycera atra MacFarland, 1905	CASIZ170506a	California	JX274084	JX274052
Polycera atra MacFarland, 1905	CASIZ170506b	California	JX274085	JX274053
Polycera aurantiomarginata García-Gómez & Bobo, 1984	-	Spain, Cadiz	AJ223274	AJ225199
Polycera aurantiomarginata García-Gómez & Bobo, 1984	MNCN15.05/55483	Morocco, Aghroud	-	JX274037
Polycera aurantiomarginata García-Gómez & Bobo, 1984	MNCN15.05/55492	Morocco, Aghroud	JX274068	JX274038
Polycera aurantiomarginata García-Gómez & Bobo, 1984	MNCN15.05/55490	Morocco, Aghroud	JX274069	JX274039
Polycera capensis Quoy & Gaimard, 1824	CASIZ176907	South Africa, Western Cape province	HM162687	HM162597
Polycera capensis Quoy & Gaimard, 1824	CASIZ176280	South Africa, Cape province	JX274091	-
Polycera capensis Quoy & Gaimard, 1824	CASIZ176375	South Africa, Western Cape Province	JX274092	JX274058
Polycera cf *capensis*	MNCN15.05/ Wales	55470Australia, New South	JX274083	JX274051
Polycera capitata (Alder & Hancock, 1854)	002	UK	**MZ425305**	**MZ420395**
Polycera capitata (Alder & Hancock, 1854)	003	UK	**MZ425306**	**MZ420396**
Polycera capitata (Alder & Hancock, 1854)	ZMMU Op-764	UK	**MZ425334**	**MZ420423**

Polycera capitata (Alder & Hancock, 1854)	ZMMU Op-765	UK	**MZ425335**	**MZ420424**
Polycera capitata (Alder & Hancock, 1854)	013	UK	**MZ425307**	**MZ420397**
Polycera capitata (Alder & Hancock, 1854)	016	UK	**MZ425308**	**MZ420398**
Polycera capitata (Alder & Hancock, 1854)	017	UK	**MZ425309**	**MZ420399**
Polycera capitata (Alder & Hancock, 1854)	018	Netherlands	**MZ425310**	**MZ420400**
Polycera capitata (Alder & Hancock, 1854)	021	UK	**MZ425311**	**MZ420401**
Polycera capitata (Alder & Hancock, 1854)	025	UK	**MZ425312**	**MZ420402**
Polycera capitata (Alder & Hancock, 1854)	029	Netherlands	**MZ425313**	**MZ420403**
Polycera capitata (Alder & Hancock, 1854)	031	UK	**MZ425314**	**MZ420404**
Polycera capitata (Alder & Hancock, 1854)	ZMMU Op-766	UK	**MZ425336**	**MZ420425**
Polycera capitata (Alder & Hancock, 1854)	034	Netherlands	**MZ425315**	**MZ420405**
Polycera capitata (Alder & Hancock, 1854)	037	UK	**MZ425316**	**MZ420406**
Polycera capitata (Alder & Hancock, 1854)	039	UK	**MZ425317**	**MZ420407**
Polycera capitata (Alder & Hancock, 1854)	042	UK	**MZ425318**	**MZ420408**
Polycera capitata (Alder & Hancock, 1854)	043	UK	**MZ425319**	**MZ420409**
Polycera capitata (Alder & Hancock, 1854)	050	UK	**MZ425320**	**MZ420410**
Polycera capitata (Alder & Hancock, 1854)	052	UK	**MZ425321**	**MZ420411**
Polycera capitata (Alder & Hancock, 1854)	053	UK	**MZ425322**	**MZ420412**
Polycera capitata (Alder & Hancock, 1854)	056	UK	**MZ425323**	**MZ420413**
Polycera capitata (Alder & Hancock, 1854)	060	Netherlands	**MZ425324**	**MZ420414**
Polycera capitata (Alder & Hancock, 1854)	061	UK	**MZ425325**	**MZ420415**
Polycera capitata (Alder & Hancock, 1854	062	UK	**MZ425326**	**MZ420416**
Polycera capitata (Alder & Hancock, 1854)	065	UK	**MZ425327**	**MZ420417**
Polycera capitata (Alder & Hancock, 1854)	067	UK	**MZ425328**	**-**
Polycera capitata (Alder & Hancock, 1854)	068	Netherlands	**MZ425329**	**MZ420418**
Polycera capitata (Alder & Hancock, 1854)	070	Netherlands	**MZ425330**	**MZ420419**

Polycera capitata (Alder & Hancock, 1854)	072	Netherlands	**MZ425331**	**MZ420420**
Polycera capitata (Alder & Hancock, 1854)	074	UK	**MZ425332**	**MZ420421**
Polycera capitata (Alder & Hancock, 1854)	075	Netherlands	**MZ425333**	**MZ420422**
Polycera capitata (Alder & Hancock, 1854)	ZMMU Op-769	Ireland	**MZ425337**	-
Polycera capitata (Alder & Hancock, 1854)	ZMMU Op-770	Ireland	**MZ425338**	-
Polycera capitata (Alder & Hancock, 1854) *Polycera "norvegica"*	ZMBN 125917	Norway: Herdla, Askøy, Bergen, Vestland	MT477918	-
Polycera capitata (Alder & Hancock, 1854) *Polycera "norvegica"*	ZMBN 126023	Norway: Uthaug, Ørland, Trøndelag	MT477917	-
Polycera capitata (Alder & Hancock, 1854) *Polycera "norvegica"*	ZMBN 127486	Norway: Tingelsædet, Egersund, Rogaland	MT477916	-
Polycera capitata (Alder & Hancock, 1854) *Polycera "norvegica"*	ZMBN 126025	Norway: Uthaug, Ørland, Trøndelag	MT477914	-
Polycera capitata (Alder & Hancock, 1854) *Polycera "norvegica"*	ZMBN 125492	Norway: Tingelsædet, Egersund, Rogaland	MT477926	-
Polycera capitata (Alder & Hancock, 1854) *Polycera "norvegica"*	ZMBN 126024	Norway: Uthaug, Ørland, Trøndelag	MT477922	-
Polycera capitata (Alder & Hancock, 1854) *Polycera "norvegica"*	ZMBN 125855 P45	Norway: Legern, Haugesund, Rogaland	MT477921	-
Polycera capitata (Alder & Hancock, 1854) *Polycera "norvegica"*	ZMBN 125855 P46	Norway: Legern, Haugesund, Rogaland	MT477924	-
Polycera capitata (Alder & Hancock, 1854) *Polycera "norvegica"*	ZMBN 125493	Norway: Sandholmane, Haugesund, Rogaland	MT477925	-
Polycera capitata (Alder & Hancock, 1854) *Polycera "norvegica"*	ZMBN 125881 P48	Norway: Sandholmane, Haugesund, Rogaland	MT477928	-
Polycera capitata (Alder & Hancock, 1854) *Polycera "norvegica"*	ZMBN 125881 P49	Norway: Sandholmane, Haugesund, Rogaland	MT477919	-
Polycera capitata (Alder & Hancock, 1854) *Polycera "norvegica"*	ZMBN 125881 P50	Norway: Sandholmane, Haugesund, Rogaland	MT477923	-
Polycera capitata (Alder & Hancock, 1854) *Polycera "norvegica"*	ZMBN 125881 P51	Norway: Sandholmane, Haugesund, Rogaland	MT477912	-
Polycera capitata (Alder & Hancock, 1854) *Polycera "norvegica"*	ZMBN 127607	Norway: Skeisvika, Hundvåg, Stavanger, Rogaland	MT477920	-
Polycera capitata (Alder &	ZMBN 127608	Norway: Skeisvika,	MT477927	-

Hancock, 1854) *Polycera "norvegica"*		Hundvåg, Stavanger, Rogaland		
Polycera capitata (Alder & Hancock, 1854) *Polycera "norvegica"*	ZMBN 127664	Norway: Seløysundet, Espegrend, Bergen, Vestland	MT477913	-
Polycera capitata (Alder & Hancock, 1854) *Polycera "norvegica"*	ZMBN 106115	Norway: Steingardsvika, Espegrend, Bergen, Vestland	NBMM034-15	-
Polycera faeroensis Lemche, 1929	028	Netherlands	**MZ425339**	**MZ420426**
Polycera faeroensis Lemche, 1929	ZMMU Op-774	UK	**MZ425340**	**MZ420427**
Polycera cf. *hedgpethi* Er. Marcus, 1964	MNCN15.05/55493	Morocco, Aghroud	JX274086	-
Polycera quadrilineata (O. F. Müller, 1776)	005	UK	**MZ425341**	**MZ420428**
Polycera quadrilineata (O. F. Müller, 1776)	014	Netherlands	-	**MZ420429**
Polycera quadrilineata (O. F. Müller, 1776)	019	UK	**MZ425342**	**MZ420430**
Polycera quadrilineata (O. F. Müller, 1776)	027	Netherlands	**MZ425343**	**MZ420431**
Polycera quadrilineata (O. F. Müller, 1776)	030	UK	**MZ425344**	**MZ420432**
Polycera quadrilineata (O. F. Müller, 1776)	038	UK	-	**MZ420433**
Polycera quadrilineata (O. F. Müller, 1776)	040	Netherlands	-	**MZ420434**
Polycera quadrilineata (O. F. Müller, 1776)	ZMMU Op-756	UK	**MZ425366**	**MZ420457**
Polycera quadrilineata (O. F. Müller, 1776)	044	Netherlands	**MZ425345**	**MZ420435**
Polycera quadrilineata (O. F. Müller, 1776)	045	Netherlands	**MZ425346**	**MZ420436**
Polycera quadrilineata (O. F. Müller, 1776)	048	Netherlands	**MZ425347**	**MZ420437**
Polycera quadrilineata (O. F. Müller, 1776)	ZMMU Op-757	UK	**MZ425367**	**MZ420458**
Polycera quadrilineata (O. F. Müller, 1776)	054	UK	**MZ425348**	**MZ420438**
Polycera quadrilineata (O. F. Müller, 1776)	055	Germany	**MZ425349**	**MZ420439**
Polycera quadrilineata (O. F. Müller, 1776)	057	Sweden	-	**MZ420440**
Polycera quadrilineata (O. F. Müller, 1776)	059	Netherlands	-	**MZ420441**
Polycera quadrilineata (O. F. Müller, 1776)	064	Netherlands	**MZ425350**	**MZ420442**
Polycera quadrilineata (O. F. Müller, 1776)	066	UK	**MZ425351**	**-**
Polycera quadrilineata (O. F. Müller, 1776)	071	Netherlands	**MZ425352**	**MZ420443**
Polycera quadrilineata (O. F.	073	Netherlands	**MZ425353**	**MZ420444**

Müller, 1776)				
Polycera quadrilineata (O. F. Müller, 1776)	077	UK	**MZ425354**	**MZ420445**
Polycera quadrilineata (O. F. Müller, 1776)	ZMMU Op-758	UK	**MZ425368**	**MZ420459**
Polycera quadrilineata (O. F. Müller, 1776)	081	UK	**MZ425355**	**MZ420446**
Polycera quadrilineata (O. F. Müller, 1776)	083	Netherlands	**MZ425356**	**MZ420447**
Polycera quadrilineata (O. F. Müller, 1776)	088	Netherlands	**MZ425357**	**MZ420448**
Polycera quadrilineata (O. F. Müller, 1776)	094	Netherlands	**MZ425358**	**MZ420449**
Polycera quadrilineata (O. F. Müller, 1776)	095	Netherlands	**MZ425359**	**MZ420450**
Polycera quadrilineata (O. F. Müller, 1776)	101	Denmark	**MZ425360**	**MZ420451**
Polycera quadrilineata (O. F. Müller, 1776)	102	Denmark	**MZ425361**	**MZ420452**
Polycera quadrilineata (O. F. Müller, 1776)	103	Denmark	**MZ425362**	**MZ420453**
Polycera quadrilineata (O. F. Müller, 1776)	ZMMU Op-759	Denmark	**MZ425369**	**MZ420460**
Polycera quadrilineata (O. F. Müller, 1776)	105	Denmark	**MZ425363**	**MZ420454**
Polycera quadrilineata (O. F. Müller, 1776)	106	Denmark	**MZ425364**	**MZ420455**
Polycera quadrilineata (O. F. Müller, 1776)	ZMMU Op-750	Norway, Gulen	**MZ425365**	**MZ420456**
Polycera quadrilineata (O. F. Müller, 1776)	ZMMU Op-760	Portugal	**MZ425370**	-
Polycera quadrilineata (O. F. Müller, 1776)	MNCN15.05/46738	United Kingdom, Oban	EF142907	-
Polycera quadrilineata (O. F. Müller, 1776)	-	North Sea, Kattegat	-	AF249229
Polycera quadrilineata (O. F. Müller, 1776)	-	Sweden, Bohuslan	AJ223275	AJ225200
Polycera quadrilineata (O. F. Müller, 1776)	MNCN15.05/55462	Sweden, Gothenborg	JX274070	JX274041
Polycera quadrilineata (O. F. Müller, 1776)	MNCN15.05/55465	Sweden, Gothenborg	JX274071	JX274042
Polycera quadrilineata (O. F. Müller, 1776)	MNCN15.05/55456	Sweden, Gothenborg	JX274072	JX274043
Polycera quadrilineata (O. F. Müller, 1776)	MNCN15.05/55466	Sweden, Gothenborg	JX274073	JX274044
Polycera quadrilineata (O. F. Müller, 1776)	MNCN15.05/55463	Sweden, Gothenborg	JX274074	JX274045
Polycera quadrilineata (O. F. Müller, 1776)	MNCN15.05/55459	Sweden, Gothenborg	JX274077	JX274046
Polycera quadrilineata (O. F. Müller, 1776)	MNCN15.05/55455	Sweden, Gothenborg	JX274079	JX274047
Polycera quadrilineata (O. F.	ZMBN 125859	Norway: Flatholmen,	MT477953	-

Müller, 1776)		Haugesund, Rogaland		
Polycera quadrilineata (O. F. Müller, 1776)	ZMBN 125613	Norway: Brattøya, Kristiansund, Møre and Romsdal	MT477972	-
Polycera quadrilineata (O. F. Müller, 1776)	ZMBN 125688	Norway: Hafrsfjord, Sola, Stavanger, Rogaland	MT477945	-
Polycera quadrilineata (O. F. Müller, 1776)	ZMBN 125971	Norway: Breidvika, Drotningsvik, Bergen, Vestland	MT477933	-
Polycera quadrilineata (O. F. Müller, 1776)	ZMBN 125032	Norway: Seløysundet, Espegrend, Bergen, Vestland	MT477952	-
Polycera quadrilineata (O. F. Müller, 1776)	ZMBN 125603	Norway: Brattøya, Kristiansund, Møre and Romsdal	MT477977	-
Polycera quadrilineata (O. F. Müller, 1776)	ZMBN 125906	Norway: Sletta, Haugesund, Rogaland	MT477964	-
Polycera quadrilineata (O. F. Müller, 1776)	ZMBN 127491	Norway: Tingelsædet, Egersund, Rogaland	MT477954	-
Polycera quadrilineata (O. F. Müller, 1776)	ZMBN 127476	Norway: Tingelsædet, Egersund, Rogaland	MT477950	-
Polycera quadrilineata (O. F. Müller, 1776)	ZMBN 127512	Norway: Litle Svetlingen, Egersund, Rogaland	MT477930	-
Polycera quadrilineata (O. F. Müller, 1776)	ZMBN 127511	Norway: Litle Svetlingen, Egersund, Rogaland	MT477971	-
Polycera quadrilineata (O. F. Müller, 1776)	ZMBN 127510	Norway: Litle Svetlingen, Egersund, Rogaland	MT477936	-
Polycera quadrilineata (O. F. Müller, 1776)	ZMBN 127487	Norway: Tingelsædet, Egersund Rogaland	MT477949	-
Polycera quadrilineata (O. F. Müller, 1776)	ZMBN 125988	Norway: Drågsvågen, Førde, Sveio, Vestland	MT477934	-
Polycera quadrilineata (O. F. Müller, 1776)	ZMBN 127488	Norway: Tingelsædet, Egersund, Rogaland	MT477960	-
Polycera quadrilineata (O. F. Müller, 1776)	ZMBN 127513	Norway: Litle Svetlingen, Egersund, Rogaland	MT477970	-
Polycera quadrilineata (O. F. Müller, 1776)	ZMBN 125635 P19	Norway: Brattøya, Kristiansund, Møre and Romsdal	MT477974	-
Polycera quadrilineata (O. F. Müller, 1776)	ZMBN 125658 P20	Norway: Brattøya, Kristiansund, Møre and Romsdal	MT477929	-
Polycera quadrilineata (O. F. Müller, 1776)	ZMBN 125658 P21	Norway: Brattøya, Kristiansund, Møre and Romsdal	MT477963	-
Polycera quadrilineata (O. F. Müller, 1776)	ZMBN 125658 P22	Norway: Brattøya, Kristiansund, Møre and Romsdal	MT477962	-

Polycera quadrilineata (O. F. Müller, 1776)	ZMBN 125688	Norway: Hafrsfjord, Sola, Stavanger, Rogaland	MT477946	-
Polycera quadrilineata (O. F. Müller, 1776)	ZMBN 125033	Norway: Seløysundet, Espegrend, Bergen, Vestland	MT477973	-
Polycera quadrilineata (O. F. Müller, 1776)	ZMBN 125635 P27	Norway: Brattøya, Kristiansund, Møre and Romsdal	MT477935	-
Polycera quadrilineata (O. F. Müller, 1776)	ZMBN 87937	Portugal: Azores, São Miguel Island	MT477940	-
Polycera quadrilineata (O. F. Müller, 1776)	ZMBN 87942	Portugal: Azores, São Miguel Island	MT477939	-
Polycera quadrilineata (O. F. Müller, 1776)	ZMBN 87925	Portugal: Azores, São Miguel Island	MT477938	-
Polycera quadrilineata (O. F. Müller, 1776)	ZMBN 97198	Portugal: Azores, Graciosa Island	MT477941	-
Polycera quadrilineata (O. F. Müller, 1776)	ZMBN 127481	Norway: Tingelsædet, Egersund, Rogaland	MT477944	-
Polycera quadrilineata (O. F. Müller, 1776)	ZMBN 127492	Norway: Tingelsædet, Egersund, Rogaland	MT477937	-
Polycera quadrilineata (O. F. Müller, 1776)	ZMBN 127509	Norway: Litle Svetlingen, Egersund, Rogaland	MT477959	-
Polycera quadrilineata (O. F. Müller, 1776)	ZMBN 125578	Norway: Drøbak, Frogn, Viken	MT477957	-
Polycera quadrilineata (O. F. Müller, 1776)	ZMBN 125689	Norway: Egersund havn, Rogaland	MT477961	-
Polycera quadrilineata (O. F. Müller, 1776)	ZMBN 125636	Norway: Nordsundet, Kristiansund, Møre and Romsdal	MT477965	-
Polycera quadrilineata (O. F. Müller, 1776)	ZMBN 127587	Norway: Drøbak, Frogn, Viken	MT477932	-
Polycera quadrilineata (O. F. Müller, 1776)	ZMBN 127600	Norway: Drøbak, Frogn, Viken	MT477943	-
Polycera quadrilineata (O. F. Müller, 1776)	ZMBN 127626	Norway: Engøy, Stavanger, Rogaland	MT477969	-
Polycera quadrilineata (O. F. Müller, 1776)	ZMBN 127631	Norway: Engøy, Stavanger, Rogaland	MT477958	-
Polycera quadrilineata (O. F. Müller, 1776)	ZMBN 127633	Norway: Engøy, Stavanger, Rogaland	MT477968	-
Polycera quadrilineata (O. F. Müller, 1776)	ZMBN 127685	Norway: Turøy, Skitholmen, Bergen, Vestland	MT477955	-
Polycera quadrilineata (O. F. Müller, 1776)	ZMBN 127689	Norway: Turøy, Myrbærholmen, Bergen, Vestland	MT477967	-
Polycera quadrilineata (O. F. Müller, 1776)	ZMBN 127690	Norway: Turøy, Myrbærholmen, Bergen, Vestland	MT477966	-
Polycera quadrilineata (O. F. Müller, 1776)	ZMBN 127682	Norway: Turøy, Skitholmen, Bergen,	MT477956	-

		Vestland		
Polycera quadrilineata (O. F. Müller, 1776)	ZMBN 127678	Norway: Turøy, Skitholmen, Bergen, Vestland	MT477948	-
Polycera quadrilineata (O. F. Müller, 1776)	ZMBN 127683	Norway: Turøy, Skitholmen, Bergen, Vestland	MT477951	-
Polycera quadrilineata (O. F. Müller, 1776)	ZMBN 127681	Norway: Turøy, Skitholmen, Bergen, Vestland	MT477976	-
Polycera quadrilineata (O. F. Müller, 1776)	ZMBN 127676	Norway: Turøy, Skitholmen, Bergen, Vestland	MT477931	-
Polycera quadrilineata (O. F. Müller, 1776)	P73	Mediterranean Spain: Mataró, Catalonia	MT477947	-
Polycera quadrilineata (O. F. Müller, 1776)	P74	Mediterranean Spain: Roses, Catalonia	MT477942	-
Polycera kernowensis sp.nov.	001	UK	**MZ425371**	**MZ420461**
Polycera kernowensis sp.nov.	ZMMU Op-775	Portugal	**MZ425400**	**MZ420490**
Polycera kernowensis sp.nov.	006	UK	**MZ425372**	**MZ420462**
Polycera kernowensis sp.nov.	009	UK	**MZ425373**	**MZ420463**
Polycera kernowensis sp.nov.	010	UK	**MZ425374**	**MZ420464**
Polycera kernowensis sp.nov.	011	UK	**MZ425375**	**MZ420465**
Polycera kernowensis sp.nov.	012	Netherlands	**MZ425376**	**MZ420466**
Polycera kernowensis sp.nov.	ZMMU Op-755	UK	**MZ425399**	**MZ420489**
Polycera kernowensis sp.nov.	020	Netherlands	**MZ425377**	**MZ420467**
Polycera kernowensis sp.nov.	022	UK	**MZ425378**	**MZ420468**
Polycera kernowensis sp.nov.	023	UK	**MZ425379**	**MZ420469**
Polycera kernowensis sp.nov.	024	UK	**MZ425380**	**MZ420470**
Polycera kernowensis sp.nov.	026	UK	**MZ425381**	**MZ420471**
Polycera kernowensis sp.nov.	032	UK	**MZ425382**	**MZ420472**
Polycera kernowensis sp.nov.	035	UK	**MZ425383**	**MZ420473**
Polycera kernowensis sp.nov.	036	UK	**MZ425384**	**MZ420474**
Polycera kernowensis sp.nov.	046	UK	**MZ425385**	**MZ420475**
Polycera kernowensis sp.nov.	ZMMU Op-776	UK	**MZ425401**	**MZ420491**
Polycera kernowensis sp.nov.	049	UK	**MZ425386**	**MZ420476**
Polycera kernowensis sp.nov.	058	UK	**MZ425387**	**MZ420477**
Polycera kernowensis sp.nov.	063	UK	**MZ425388**	**MZ420478**
Polycera kernowensis sp.nov.	069	UK	**MZ425389**	**MZ420479**
Polycera kernowensis sp.nov.	076	UK	**MZ425390**	**MZ420480**
Polycera kernowensis sp.nov.	079	UK	**MZ425391**	**MZ420481**
Polycera kernowensis sp.nov.	085	UK	**MZ425392**	**MZ420482**
Polycera kernowensis sp.nov.	086	UK	**MZ425393**	**MZ420483**
Polycera kernowensis sp.nov.	087	UK	**MZ425394**	**MZ420484**
Polycera kernowensis sp.nov.	089	UK	**MZ425395**	**MZ420485**
Polycera kernowensis sp.nov.	091	UK	**MZ425396**	**MZ420486**
Polycera kernowensis sp.nov.	093	UK	**MZ425397**	**MZ420487**
Polycera kernowensis sp.nov.	100	UK	**MZ425398**	**MZ420488**
Polycera kernowensis sp.nov.	MNCN15.05/55503.1	Portugal, Estacada	JX274088	-
Polycera kernowensis sp.nov.	MNCN15.05/55503.2	Portugal, Estacada	JX274089	JX274056
Polycera tricolor Robilliard, 1971	CASIZ76438a	California, San Francisco Estuary	JX274087	JX274054

Polycera tricolor Robilliard, 1971	CASIZ76438b	California, San Francisco Estuary	-	JX274055
Polycera sp. A	CASIZ176169	South Africa, Western Cape Province	JX274081	JX274049
Polycera sp. A	CASIZ176387	South Africa, Eastern Cape Province	JX274082	JX274050
Polycera sp. B	CASIZ176795	Hawaii, Maui	JX274093	-
Polycera sp. C	CASIZ120773	Pacific Ocean, Marshall Islands	JX274090	JX274057
Polycerella emertoni A. E. Verrill, 1880	-	Spain, Cadiz	AJ223273	AJ225198
Polycerella emertoni A. E. Verrill, 1880	MNCN15.05/55480	Spain, Cadiz	JX274095	JX274060
Polycerella emertoni A. E. Verrill, 1880	MNCN15.05/55482a	Spain, Cadiz	JX274098	JX274061
Polycerella emertoni A. E. Verrill, 1880	MNCN15.05/55482b	Spain, Cadiz	JX274099	JX274062
Tambja crioula Pola, Padula, Gosliner & Cervera, 2014	CASIZ:180377	Cape Verde	KJ999222	KJ999201
Tambja kava Pola, Padula, Gosliner & Cervera, 2014	CASIZ:178792	Vanuatu	KJ999224	KJ999203
Tambja marbellensis Schick & Cervera, 1998	CASIZ:180379	Portugal	HM162689	HM162599
Thecacera pennigera (Montagu, 1813)	-	Spain, Cadiz	AJ223277	AJ225202
Thecacera cf. *pennigera* (Montagu, 1813)	CASIZ176285	South Africa, Cape province	JX274094	JX274059
Thecacera picta Baba, 1972	-	USA: California	KP871652	KP871701
Triopha catalinae (Cooper, 1863)	CASIZ:170648	USA: California	HM162690	HM162600
Triopha maculata MacFarland, 1905	CASIZ:181556	USA: California	HM162691	HM162601

Supplementary information Table S2. The data were used for statistical analysis (the data for white and orange colour variants highlighted in bold).

Polycera quadrilineata

№	Rhinophoral lamellae (number)	Frontal veil appendages (number)	Gills (number)	Body length (mm)
1	10	4	5	6,2
**2**	**16**	**6**	**-**	**17,1**
**3**	**10**	**4**	**6**	**7,7**
4	11	6	-	18
5	11	4	7	8,1
**6**	**9**	**4**	**7**	**7,3**
7	13	5	-	17,4
8	9	4	6	8,9
9	14	4	-	16,2
10	13	4	-	16,3
**11**	**8**	**4**	**-**	**11,2**
**12**	**11**	**4**	**6**	**6,7**
13	9	4	5	6,1
14	12	4	-	11,1
15	7	4	-	6,5
**16**	**17**	**3**	**-**	**9,6**
**17**	**11**	**4**	**6**	**6,1**
**18**	**11**	**6**	**6**	**3**
**19**	**11**	**4**	**6**	**8,9**
**20**	**16**	**6**	**-**	**9,3**
21	13	6	-	16
22	10	6	6	10
**23**	**8**	**4**	**7**	**6**
24	11	4	7	20
25	10	6	5	12
26	9	4	6	9
27	10	4	7	15
**28**	**11**	**4**	**9**	**26**
**29**	**14**	**6**	**8**	**12**
**30**	**14**	**4**	**8**	**12**
31	16	4	7	-
**32**	**11**	**6**	**7**	**-**
33	10	4	6	-
34	11	4	8	-
35	12	6	6	-
36	11	4	10	-
37	11	4	6	-
38	10	4	6	-
**39**	**11**	**4**	**9**	**-**
**40**	**17**	**4**	**7**	**-**

Polycera capitata

№	Rhinophoral lamellae (number)	Frontal veil appendages (number)	Gills (number)	Body length (mm)
**1**	**8**	**4**	**6**	**6,2**
**2**	**8**	**4**	**6**	**5,4**
**3**	**9**	**5**	**7**	**6**
4	8	4	7	6,5
5	7	4	6	3,2
**6**	**8**	**4**	**8**	**6,5**
7	8	4	7	5,2
**8**	**8**	**4**	**9**	**7,2**
**9**	**10**	**4**	**7**	**5,8**
**10**	**8**	**4**	**-**	**5,4**
**11**	**8**	**6**	**7**	**6,9**
**12**	**7**	**4**	**6**	**4,9**
13	8	4	6	6,4
**14**	**8**	**4**	**7**	**7,3**
15	8	4	6	6,5
16	7	4	6	6,5
**17**	**7**	**4**	**7**	**5,2**
18	7	4	7	4,9
19	10	4	6	6,3
20	9	4	6	5,6
**21**	**7**	**4**	**7**	**6**
**22**	**10**	**4**	**7**	**7,2**
23	6	4	7	5,9
24	9	4	7	7,9
25	9	6	6	10
**26**	**9**	**6**	**8**	**13**
27	10	4	8	16
28	9	4	8	14
29	8	5	6	12
30	8	4	8	11
**31**	**7**	**4**	**4**	**-**
**32**	**7**	**4**	**5**	**8**
33	7	4	6	5
34	7	4	6	-

Polycera faeroensis

№	Rhinophoral lamellae (number)	Frontal veil appendages (number)	Gills (number)	Body length (mm)
**1**	**18**	**9**	**10**	**30**
**2**	**10**	**8**	**-**	**21,1**
**3**	**21**	**8**	**6**	**18**
**4**	**15**	**10**	**7**	**25**
**5**	**19**	**10**	**7**	**21**
**6**	**19**	**9**	**6**	**30**
**7**	**18**	**9**	**7**	**25**

8	20	10	6	30
9	23	7	6	25
10	21	10	7	23
11	17	12	6	30
12	17	7	6	30
13	19	10	6	22
14	17	13	6	25
15	15	10	6	-
16	20	8	7	-
17	18	8	7	-

***Polycera kernowensis* sp. nov.**

№	Rhinophoral lamellae (number)	Frontal veil appendages (number)	Gills (number)	Body length (mm)
1	14	8	4	4,4
2	15	8	5	3,9
3	14	8	3	4,5
4	16	7	5	6,7
5	14	8	5	5,6
6	13	8	4	5
7	11	8	-	13,9
8	14	8	6	5,6
9	13	8	4	6,1
10	15	7	5	8,5
11	14	7	5	6,8
12	11	8	3	3,5
13	12	7	3	3,5
14	15	7	6	6,1
15	15	8	5	6,5
16	14	8	5	7,1
17	13	8	4	5,5
18	14	8	5	6,2
19	16	7	-	7,5
20	16	8	5	7,3
21	14	8	4	3,7
22	13	7	3	4,1
23	10	6	3	3,9
24	14	8	3	5,5
25	14	8	4	5,5
26	14	8	4	5,8
27	16	8	6	5,4
28	14	6	4	6,7
29	16	7	6	20
30	22	8	6	20
